# Plasma metabolomics provides new insights into the relationship between metabolites and outcomes and left ventricular remodeling of coronary artery disease

**DOI:** 10.1186/s13578-022-00863-x

**Published:** 2022-10-14

**Authors:** Qian Zhu, Min Qin, Zixian Wang, Yonglin Wu, Xiaoping Chen, Chen Liu, Qilin Ma, Yibin Liu, Weihua Lai, Hui Chen, Jingjing Cai, Yemao Liu, Fang Lei, Bin Zhang, Shuyao Zhang, Guodong He, Hanping Li, Mingliang Zhang, Hui Zheng, Jiyan Chen, Min Huang, Shilong Zhong

**Affiliations:** 1 Department of Pharmacy, Guangdong Provincial People’s Hospital, Guangdong Academy of Medical Sciences, Guangzhou, 510080 Guangdong China; 2 Guangdong Provincial Key Laboratory of Coronary Heart Disease Prevention, Guangdong Cardiovascular Institute, Guangdong Provincial People’s Hospital, Guangdong Academy of Medical Sciences, Guangzhou, 510080 Guangdong China; 3 School of Medicine, South China University of Technology, Guangzhou, 510080 Guangdong China; 4 Department of Clinical Pharmacology, Xiangya Hospital, Central South University, Changsha, 410008 Hunan China; 5 Department of Cardiology, The First Affiliated Hospital of Sun Yat-Sen University, Guangzhou, 510080 Guangdong China; 6 Department of Cardiology, Xiangya Hospital, Central South University, Changsha, 410008 Hunan China; 7 Institute of Model Animal, Wuhan University, Wuhan, 430072 Hubei China; 8 Department of Pharmacy, Guangzhou Red Cross Hospital, Jinan University, Guangzhou, 510220 Guangdong China; 9 Wuhan Metware Biotechnology Co., Ltd., Wuhan, 430000 Hubei China; 10 Institute of Clinical Pharmacology, School of Pharmaceutical Sciences, Sun Yat-Sen University, Guangzhou, 510006 Guangdong China

**Keywords:** Metabolomics, Coronary artery disease, Death, Major adverse cardiovascular events, Metabolic signature, Left ventricular remodeling, Mendelian randomisation

## Abstract

**Background:**

Coronary artery disease (CAD) is a metabolically perturbed pathological condition. However, the knowledge of metabolic signatures on outcomes of CAD and their potential causal effects and impacts on left ventricular remodeling remains limited. We aim to assess the contribution of plasma metabolites to the risk of death and major adverse cardiovascular events (MACE) as well as left ventricular remodeling.

**Results:**

In a prospective study with 1606 Chinese patients with CAD, we have identified and validated several independent metabolic signatures through widely-targeted metabolomics. The predictive model respectively integrating four metabolic signatures (dulcitol, β-pseudouridine, 3,3ʹ,5-Triiodo-l-thyronine, and kynurenine) for death (AUC of 83.7% vs. 76.6%, positive IDI of 0.096) and metabolic signatures (kynurenine, lysoPC 20:2, 5-methyluridine, and l-tryptophan) for MACE (AUC of 67.4% vs. 59.8%, IDI of 0.068) yielded better predictive value than trimethylamine N-oxide plus clinical model, which were successfully applied to predict patients with high risks of death (P = 0.0014) and MACE (P = 0.0008) in the multicenter validation cohort. Mendelian randomisation analysis showed that 11 genetically inferred metabolic signatures were significantly associated with risks of death or MACE, such as 4-acetamidobutyric acid, phenylacetyl-l-glutamine, tryptophan metabolites (kynurenine, kynurenic acid), and modified nucleosides (β-pseudouridine, 2-(dimethylamino) guanosine). Mediation analyses show that the association of these metabolites with the outcomes could be partly explained by their roles in promoting left ventricular dysfunction.

**Conclusions:**

This study provided new insights into the relationship between plasma metabolites and clinical outcomes and its intermediate pathological process left ventricular dysfunction in CAD. The predictive model integrating metabolites can help to improve the risk stratification for death and MACE in CAD. The metabolic signatures appear to increase death or MACE risks partly by promoting adverse left ventricular dysfunction, supporting potential therapeutic targets of CAD for further investigation.

**Graphical Abstract:**

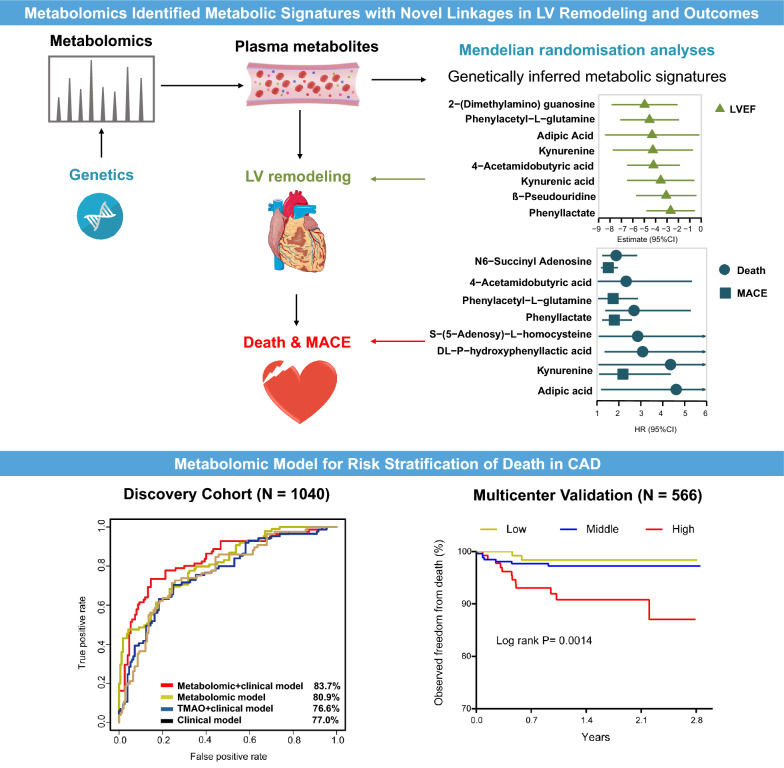

**Supplementary Information:**

The online version contains supplementary material available at 10.1186/s13578-022-00863-x.

## Background

Coronary artery disease (CAD) imposes a major burden on modern society with annual morbidity and mortality comparable to cancer [1–3]. Despite the advances in pharmaceutical and operative treatments, the mortality of CAD remains unacceptably high. It is well-known that significant left ventricular (LV) remodeling is a severe and common issue in CAD [4], which contributes to the development of heart failure and the noticeably elevated risk of mortality and cardiovascular events [5, 6]. However, available molecular biomarkers in reflecting progressive worsening cardiac function and the long-term outcomes during CAD progression are still limited [7–9]. Incorporating novel molecular biomarkers to explain detailed molecular processes and pathophysiological mechanisms involving adverse outcomes, may improve early risk stratification and indicate novel targets in preventive therapies for CAD patients.

CAD is a metabolically perturbed pathological condition [10, 11]. Plasma metabolites reflect a functional output for genetic makeup and environmental exposure to disease phenotypes [12, 13]. Metabolomics profiling can assist in shedding light on underlying molecular mechanisms involving the pathophysiology of disease states and support personalized risk prediction for disease development and prognosis [13]. In the setting of prevalent CAD, emerging metabolomic profiling has begun to illustrate specific molecular signatures relating to CAD characterization [10]. Besides, previous studies have also identified a few metabolic markers for cardiovascular events or all-cause mortality [14–18], mainly in Westerners or free of cardiovascular disease. Moreover, researchers have indicated that different study populations may lead to different molecular signatures and conflicting findings [19–22]. While most metabolomic studies have little focus on further explanation of the causal effects and potential pathophysiologic mechanisms of metabolic markers. The mechanism promoting death and major adverse events (MACE) occurrence in CAD patients are heterogeneous, the potential pathological mechanism of adverse LV remodeling during CAD progression has become one of the hot issues [23]. However, few studies have examined the association of metabolites with clinical outcomes and cardiac remodeling in CAD, and the potential causal roles that the spectrum of metabolites play during the disease progression, before clinical endpoints onset, is thus unclear.

Given the fact that metabolism has been closely implicated in CAD pathogenesis and development, there is a need to identify novel metabolic signatures and integrate the genetic regulation of circulating metabolites to improve understanding in causality, and thus provide potential pathological insights and therapeutic targets to improve CAD survival and prognosis. Therefore, we performed a study on 1606 Chinese patients with CAD through metabolomic profiling in plasma to evaluate the contribution of metabolites to the risks of death or MACE as well as LV remodeling and then built a good prognostic model based on metabolic signatures. Moreover, we further conducted Mendelian randomisation (MR) analysis by integrating genomic data to infer the potential causal effects of metabolites, and mediation analysis to explore possible mediation effects through promoting LV remodeling.

## Results

### Patient characteristics

The baseline characteristics are listed in Table 1. We recruited 1606 patients with CAD, including 1040 patients for the discovery cohort (63.03 years, 79.62% male), and 566 multicentre patients for the multicenter validation cohort (62 years, 74.16% male). Patients with high death and MACE risks were commonly old, suffered from diabetes, high aspartate aminotransferase, SYNTAX scores, N-terminal-pro brain natriuretic peptide (proBNP) and LVMI, and low estimated glomerular filtration rate and LVEF, see Additional file 1: Table S1.

**Table 1:** Baseline characteristics in 1606 CAD patients

Characteristics	Discovery cohort (n = 1040)	Multicenter validation cohort (n = 566)	
Demographic data			
Age	63.03 ± 10.04	62.29 ± 10.18	
Sex (male)	828 (79.62)	419 (74.16)	
BMI, kg/m^2^	24.28 ± 4.79	24.06 ± 3.38	
SBP, mm Hg	130.66 ± 18.89	133.04 ± 20.29	
DBP, mm Hg	76.19 ± 11.03	76.46 ± 12.05	
Current smoking	294 (28.52)	160 (28.73)	
Family of CVD	29 (2.79)	–	
Comorbidities			
Arrhythmia	92 (8.86)	51 (9.17)	
DM	286 (27.55)	164 (29.39)	
HyperT	627 (60.35)	340 (60.93)	
Dyslipidemia	729 (72.54)	400 (74.07)	
Biomedical measurements			
ALT, U/L	27.41 ± 13.18	27.65 ± 24.56	
AST, U/L	26.64 ± 10.62	32.12 ± 55.48	
eGFR, mL/min/1.73 m^2^	94.32 ± 73.69	91.37 ± 110.84	
GLUC, mmol/L	6.74 ± 2.73	6.21 ± 3.82	
CHOL, mmol/L	4.28 ± 1.12	4.29 ± 1.77	
LDLC, mmol/L	2.58 ± 0.93	2.7 ± 1.00	
HDLC, mmol/L	0.97 ± 0.26	0.99 ± 0.25	
TRIG, mmol/L	1.62 ± 1.14	1.85 ± 1.85	
CKMB, U/L	7.48 ± 5.92	19.37 ± 52.83	
proBNP, pg/mL	774.51 ± 1597.35	1299.46 ± 4922.09	
Medications			
BB	929 (89.5)	477 (84.57)	
ACEI	660 (63.58)	286 (50.71)	
CCB	295 (28.42)	165 (30.05)	
PPI	506 (48.75)	380 (67.26)	
SYNTAX score	16.43 ± 10.74	16.45 ± 13.09	
LVEF, %	60.1 ± 11.54	59.43 ± 11.63	
LVMI, g/m^2^	122.13 ± 36.05	116.54 ± 35.08	

Data are number (%) or mean ± SD when appropriate

*SD* standard deviation, *BMI* body mass index, *SBP* systolic blood pressure, *CVD* cardiovascular disease, *DM* diabetes, *HyperT* hypertension, *ALT* alanine aminotransferase, *AST* aspartate aminotransferase, *eGFR* estimated glomerular filtration rate, *GLUC* glucose, *CHOL* cholesterol, *LDLC* low-density lipoprotein cholesterol, *HDLC* high-density lipoprotein cholesterol, *TRIG* triglyceride, *CKMB* creatine kinase MB, *proBNP* N-terminal pro brain natriuretic peptide, *BB* β-blockers, *ACEI* angiotensin converting enzyme inhibitors, *CCB* calcium channel blockers, *PPI* proton pump inhibitors, *SYNTAX* *score* Synergy between PCI with TAXUS and Cardiac Surgery score, *LVEF* left ventricular ejection fraction, *LVMI* left ventricular mass index

It is noteworthy that adverse LV remodeling manifested as low LVEF and high LVMI was strongly related to increased death or MACE risks in CAD (Additional file 2: Fig. S1), compared with other clinical characteristics including coronary lesion score. The association between characteristics with LVEF and LVMI is included in Additional file 1: Table S2.

### Metabolomic associations with the clinical outcomes

**Fig. 1:**
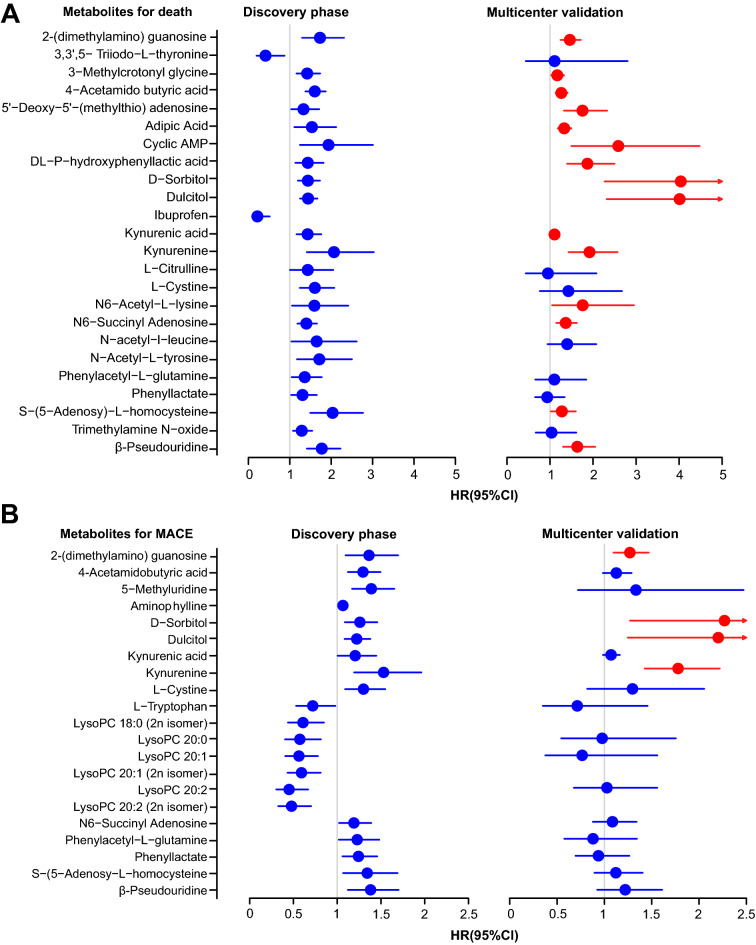
Forest plot of metabolites associated with the risks of death (**A**) and MACE (**B**). HR (hazard ratios) of metabolites in the adjusted analysis (*FDR* < 0.05) of the discovery cohort (Left) and the multicenter validation cohort (Right) after adjustment with potential confounder. HR (circles) indicate the risk of a change in each metabolite of 1 standard deviation (SD) for ease of comparison. Bars represent 95% confidence intervals. Red-coded circles and bars indicate that the metabolites were replicated in the validation cohort (*P* < 0.05)

In the discovery phase, among the 202 metabolites, we identified 35 plasma metabolites that were significantly associated with the risks of death, and 24 metabolites remained significant after adjustment for confounding factors (*FDR* < 0.05), see Fig. 1A and Additional file 1: Table S3. The most significant metabolites including 4-acetamidobutyric acid (HR, 1.60; 95% CI 1.38–1.87; *FDR* = 3.27E−08), β-pseudouridine (HR, 1.77; 95% CI 1.41–2.23; *FDR* = 9.64E−06), dulcitol (HR, 1.44; 95% CI 1.25–1.67; *FDR* = 7.10E−06), (2-(dimethylamino) guanosine (HR, 1.73; 95% CI 1.29–2.31; *FDR* = 6.73E−04), S-(5-adenosy)-l-homocysteine (HR, 2.03; 95% CI 1.49–2.77; *FDR* = 3.92E−05) and kynurenine (HR, 2.07; 95% CI 1.41–3.03; *FDR* = 6.65E−04). In the multicenter validation cohort, 15 metabolites positively associated with the death risk were reproduced, including 4-acetamidobutyric acid, β-pseudouridine, 2-(dimethylamino) guanosine, S-(5-Adenosy)-l-homocysteine, kynurenine, cyclic AMP, adipic acid, 3-methylcrotonyl glycine, 5ʹ-deoxy-5ʹ-(methylthio) adenosine, Dl-P-hydroxyphenyllactic acid, D-sorbitol, Dulcitol, kynurenic acid, *N*6-succinyl adenosine, and *N*6-acetyl-l-Lysine.

Twenty-one metabolites were significantly associated with the risks of MACE after adjustment for confounders (*FDR* < 0.05) in the discovery phase, see Fig. 1B and Additional file 1: Table S4. Notably, 4-acetamidobutyric acid (HR, 1.42; 95% CI 1.25–1.60; *FDR* = 8.87E−06) was also the most significant metabolites. During multicenter validation, 2-(dimethylamino) guanosine (HR, 1.27; 95% CI 1.10–1.47; *FDR* = 1.19E−02), dulcitol (HR, 2.20; 95% CI 1.25–3.90; *FDR* = 2.82E−02), d-sorbitol (HR, 2.27; 95% CI 1.27–4.05; *FDR* = 3.17E−02) and kynurenine (HR, 1.78; 95% CI 1.43–2.22; *FDR* = 5.88E−06) were well-replicated to be positively associated with MACE risks. 4-Acetamidobutyric acid, β-pseudouridine, kynurenic acid, and l-tryptophan were significant only before adjustment. In particular, kynurenine was validated to be robustly associated with both death and MACE risks.

### Independent metabolic signatures and optimized prediction model for clinical outcomes

To identify a minimal set of metabolites and develop a prognostic model, we firstly selected independent metabolic signatures based on the 24 metabolites associated with the risks of death from the discovery cohort, using lasso Cox regression adjusting for potential confounders (200 repeats, Table 2). Then, irrelevant metabolites were filtered out and 13 endogenous metabolites were retained, including 4-acetamidobutyric acid, dulcitol, *N*6-succinyl adenosine, l-cystine, β-pseudouridine, 2-(dimethylamino) guanosine, kynurenine, 3,3ʹ,5-triiodo-l-thyronine, d-sorbitol, DL-P-hydroxyphenyllactic acid, phenyllactate, cyclic AMP, and S-(5-Adenosy)-l-homocysteine. Besides, among the 21 metabolites associated with MACE risk, 11 endogenous metabolites were identified as independent metabolic signatures, including 4-acetamidobutyric acid, l-cystine, l-tryptophan, dulcitol, 5-methyluridine, kynurenine, phenyllactate, lysoPC 20:2, d-sorbitol, lysoPC 20:1, *N*6-succinyl adenosine.

**Table 2:** Independent metabolic signature selection using LASSO

Terms	Coefficient (β)	HR	Frequency
LASSO based signature selection for death			
Dulcitol	0.22	1.25	200
4-Acetamidobutyric acid	0.29	1.34	200
*N*6-succinyl adenosine	0.05	1.05	195
l-Cystine	0.08	1.08	191
β-Pseudouridine	0.05	1.05	173
2-(Dimethylamino) guanosine	0.02	1.02	137
Kynurenine	0.04	1.04	43
3,3ʹ,5-Triiodo-l-thyronine	− 0.12	0.89	43
d-Sorbitol	0.04	1.04	21
DL-P-hydroxyphenyllactic acid	0.02	1.03	21
Phenyllactate (PLA)	0.01	1.01	11
Cyclic AMP	0.02	1.02	5
S-(5-Adenosy)-l-homocysteine	0.02	1.02	2
LASSO based signature selection for MACE			
4-Acetamidobutyric acid	0.06	1.06	200
l-Cystine	0.06	1.06	200
l-Tryptophan	− 0.24	0.79	200
Dulcitol	0.10	1.10	200
5-Methyluridine	0.28	1.33	200
Kynurenine	0.22	1.25	200
Phenyllactate (PLA)	0.10	1.11	200
LysoPC 20:2	− 0.51	0.60	200
d-Sorbitol	0.02	1.02	199
LysoPC 20:1	− 0.04	0.96	193
*N*6-succinyl adenosine	− 0.01	0.99	2

The regression coefficients were calculated by averaging the coefficients obtained from tenfold cross-validation lasso Cox regression with 200 repeats, adjusted for 17 main clinical confounders. The confounders included age, sex, AST, eGFR, DM, HyperT, CHOL, HDLC, PPI, ACEI, BB, CCB, current smoking, family history of CVD, SYNTAX, SBP, and GLUC. The variables that appear zero times were removed and the variables left were further selected to develop a predictive model, abbreviations are as in Table 1

**Fig. 2:**
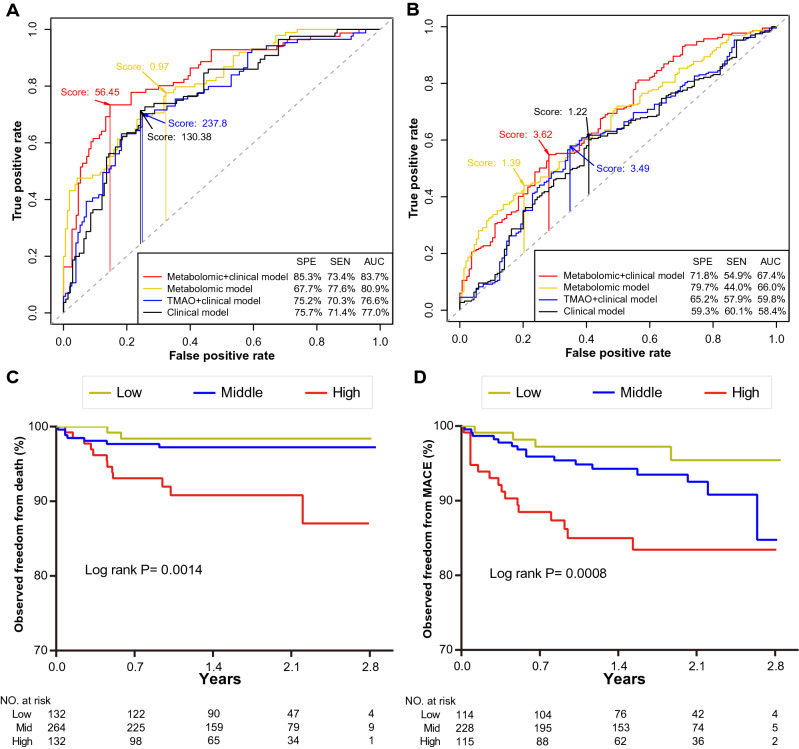
A predictive model based on metabolic signatures of death and MACE risks. ROC curves of death (**A**) and MACE (**B**) risks in the discovery cohort. Kaplan–Meier curves of the optimized predictive model (metabolomic + clinical) predicting death (**C**) and MACE (**D**) in the multicenter validation cohort between the low (< Q1), middle (≥ Ql and ≤ Q3), and high (> Q3) quartiles of hazard estimates. The optimal cutoff of the risk score was determined by calculating the highest Youden’s J value. *ROC curve* receiver–operating characteristic curve, *AUC* areas under the curve, *SPE* specificity, and *SEN* sensitivity

Then, we developed prognostic models for predicting risks of clinical endpoints in the discovery cohort, using a multivariate Cox regression model based on minimal AIC. For the risk of death, a clinical model was built based on 17 clinical risk factors, and eight factors were retained in the final model. The trimethylamine oxide (TMAO) model consisted of TMAO and five clinical variables. The metabolomic model was built only based on the metabolites screened after lasso Cox regression and finally included five metabolites (3,3ʹ,5-Triiodo-l-thyronine, β-pseudouridine, Cyclic AMP, dulcitol, and kynurenine), see Additional file 1: Table S5. The metabolomic plus clinical model combining variables of the metabolomic and clinical model, in the final included four metabolites (3,3ʹ,5-Triiodo-l-thyronine, β-pseudouridine, dulcitol, and kynurenine) and five clinical variables. The metabolomic model yielded higher predictive efficiency than the clinical model (AUC, 80.9% vs. 77.0%). Moreover, the model combining metabolomics and the clinical model for death yielded an improved predictive efficiency than the TMAO and the clinical model (Table 3), with increased AUC (83.7% vs. 76.6% vs. 77.0%, Fig. 2A), positive IDI of 0.096, continuous NRI of 0.230 and 0.121. In addition, a metabolomic model for MACE contains six metabolites (Additional file 1: Table S5) that performed well than the clinical model (AUC, 66.0 vs. 58.4%). Furthermore, adding four metabolomic variables (kynurenine, lysoPC 20:2, 5-methyluridine, and l-tryptophan) to the clinical model was also with better predictive value than that of the TMAO and the clinical model (Table 3), with increased AUC (67.4% vs. 59.8% vs. 58.4%, Fig. 2B), positive IDI of 0.068 and 0.072, and continuous NRI of 0.144 and 0.106.

**Table 3:** Model performance measures for mortality and MACE risks in the discovery phase

Predictive model	AUC	IDI (95% CI)	Continuous NRI (95% CI)
Prediction of death			
Metabolomic + clinical^a^	83.7		
Metabolomic^b^	80.9	0.072 (− 0.067 to 0.238)	0.013 (− 0.259 to 0.263)
TMAO + clinical^c^	76.6	0.096 (0.031–0.235)	0.230 (− 0.032 to 0.446)
Clinical^d^	77.0	0.096 (0.012–0.231)	0.121 (− 0.127 to 0.369)
Prediction of MACE			
Metabolomic + clinical^e^	67.4		
Metabolomic^f^	66.0	0.066 (0.005–0.124)	0.097 (−0.049 to 0.238)
TMAO + clinical^g^	59.8	0.068 (0.029–0.118)	0.144 (0.005–0.324)
Clinical^h^	58.4	0.072 (0.034–0.128)	0.106 (− 0.001–0.321)

The metabolic variables screened from LASSO, TMAO, and the 17 traditional clinical factors including age, sex, AST, eGFR, DM, HyperT, CHOL, HDLC, PPI, ACEI, BB, CCB, current smoking, family history of CVD, SYNTAX, SBP, and GLUC were input into multivariate Cox proportional hazards regression analysis to fit model, using a forward and backward stepwise process based on AIC (Akaike information criterion). The model with the smallest AIC value was considered the best and variables with P < 0.1 were retained. IDI (integrated discrimination improvement) and continuous NRI (net reclassification improvement) were calculated by comparing the Metabolomic + clinical model with TMAO + clinical and Clinical model, and the Metabolomic model with Clinical model, 95% CIs were calculated by 1000 bootstrap resampling

^a^Metabolomic + clinical model = Dulcitol, β-Pseudouridine, 3,3ʹ,5-Triiodo-l-thyronine, Kynurenine, age, current smoking, GLUC, AST, SBP

^b^Metabolomic model = Dulcitol, Kynurenine, Cyclic AMP, 3,3ʹ,5-Triiodo-l-thyronine, β-Pseudouridine

^c^TMAO model = TMAO, age, AST, current smoking, SBP, GLUC

^d^Clinical model = age, AST, HDLC, CCB, current smoking, SYNTAX, SBP, GLUC

^e^Metabolomic + clinical model = lysoPC 20:2, 5-methyluridine, kynurenine, L-tryptophan, AST, DM, PPI, SYNTAX

^f^Metabolomic model = lysoPC 20:2, 5-methyluridine, kynurenine, l-tryptophan, d-sorbitol, phenyllactate

^g^TMAO + clinical model = TMAO, AST, DM, PPI, CCB, SYNTAX

^h^Clinical model = AST, DM, PPI, CCB, SYNTAX. AUC = area under the curve, other abbreviations are as in Table 1

Subsequently, the optimized prediction model was used to estimate survival probabilities from death and MACE of each patient in the multicenter validation cohort. Survival curves showed that the model combining multiple metabolites with clinical factors could successfully differentiate patients with a low, middle, and high risk of death (log-rank test, *P* = 0.0014; Fig. 2C) and MACE (*P* = 0.0008, Fig. 2D).

### Metabolomic associations with LV remodeling

**Fig. 3:**
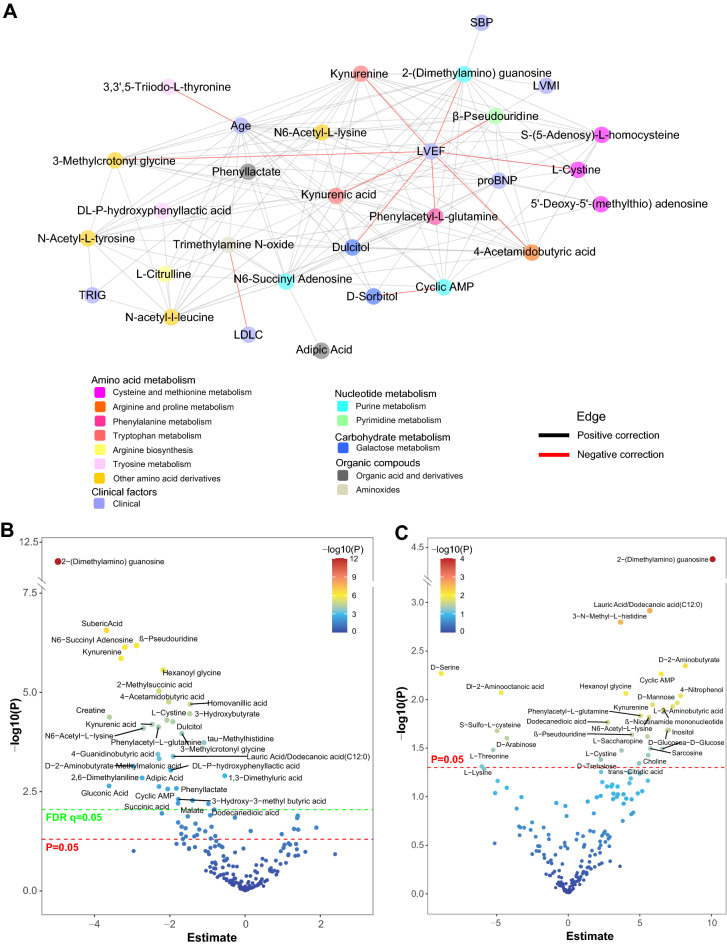
Correlation network of metabolic signatures and clinical factors, and metabolomic association with LV remodeling. **A** Correlation network of the metabolic signatures for death risk in the discovery cohort and traditional clinical factors by Spearman correlations with distant nodes of |rho|> 0.1 for clinical factors and |rho|> 0.2 for metabolites, *P* < 0.01, the rho and *P*-value were provided in Additional file 1: Table S6. Volcano plot presentation of univariate association of metabolites with LVEF (**B**) and LVMI (**C**) in a linear regression model. Metabolites with *P* < 0.05 are labeled (above the red dotted line), *P*-value cutoff equivalent to *FDR* < 0.05 are labeled (above the green dotted line), abbreviations are as Table 1

**Fig. 4:**
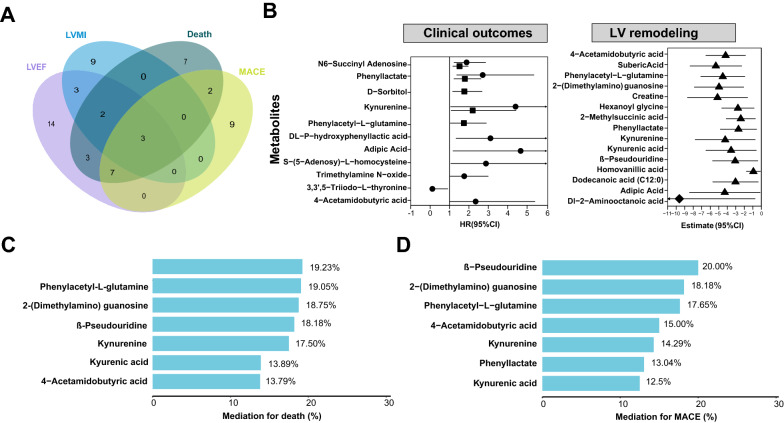
Inference of potential causality and mediation effects between metabolites, LV remodeling, and outcomes. **A** Venn plot between metabolic signatures associated with LVEF, LVMI, and risks of death and MACE in the adjusted analysis. **B** Forest plot of metabolites associated with risks of death and MACE, LVEF, and LVMI identified by Mendelian randomisation analysis. HR of death and MACE were denoted using circle and square, estimates of LVEF and LVMI were denoted using triangle and diamond, respectively. **C**, **D** The percentage of association (mediation (%)) between metabolites and outcomes (death and MACE) by LVEF using mediation analyses

Network analysis of metabolites associated with risks of death (Fig. 3A, Additional file 1: Table S6) or MACE (Additional file 1: Table S7, Additional file 2: Fig. S2) not only showed the importance of hub metabolites of 2-dimethylguanosine and kynurenine, but also important roles in reduced LVEF and increased proBNP. The relationships between metabolites with LVEF (Fig. 3B) and LVMI (Fig. 3C) in the discovery cohort were revealed, 32 metabolites were associated with LVEF after adjustment (*FDR* < 0.05), and 20 metabolites were validated during multicenter validation (*P* < 0.05, Additional file 1: Table S8). Besides, 16 metabolites were associated with LVMI and eight metabolites were validated (*P* < 0.05, Additional file 1: Table S9). Interestingly, the hub metabolites, 2-(dimethylamino) guanosine was the most significant metabolite associated with reduced LVEF and increased LVMI. Another modified nucleoside, β-pseudouridine also showed a similar finding. In particular, 15 metabolites were not only associated with increased death risks but also reduced LVEF, 10 metabolites were associated with increased MACE risks and reduced LVEF (Fig. 4A). Moreover, five metabolites were shared by increased LVMI and death risks, three metabolites were shared by increased LVMI and MACE risks. For example, 2-(dimethylamino) guanosine (estimate [SE], − 4.75 [0.69]; *FDR* = 4.72E−10), β-pseudouridine (estimate [SE], − 2.80 [0.56]; *FDR* = 9.56E−6), kynurenine (estimate [SE], − 3.15 [0.68]; *FDR* = 2.82E−05), *N*6-succinyl adenosine (estimate [SE], − 2.58 [0.64], *FDR* = 1.95E−04), 4-acetamidobutyric acid (estimate [SE], − 1.79 [0.46]; *FDR* = 3.55E−04), dulcitol (estimate [SE], − 1.67 [0.46]; *FDR* = 7.49E−04), kynurenic acid (estimate [SE], − 2.16 [0.61]; *FDR* = 9.15E−04), phenylacetyl-l-glutamine (estimate [SE], − 1.86 [0.58]; *FDR* = 2.62E−03), adipic acid (estimate [SE], − 2.30 [0.74]; *FDR* = 3.35E−03), DL-P-hydroxyphenyllactic acid (estimate [SE], − 1.41 [0.58]; *FDR* = 1.86E−02), and cyclic AMP (estimate [SE], − 1.36 [0.68]; *FDR* = 4.82E−02) were validated to be associated with reduced LVEF. Besides, 2-(dimethylamino) guanosine (estimate [SE], 7.89 [2.46]; *P* = 1.38E−03), cyclic AMP (estimate [SE], 5.27 [2.35]; *P* = 2.53E−02), kynurenine (estimate [SE], 4.88 [2.35]; *P* = 3.83E−02) and phenylacetyl-l-glutamine (estimate [SE], 4.20 [2.05]; *P* = 4.12E−02) was confirmed to be related to increased LVMI.

### Causality and mediation effects inference between metabolites, LV remodeling and clinical outcomes by MR analyses and mediation model

As secondary analyses, we performed MR analyses using genetic variants as the instrumental variables, to rule out the influences from confounders, thus providing inference of causality between the important metabolic signatures and clinical outcomes as well as LV remodeling (Fig. 4B and Additional file 1: Table S10). The MR analyses identified that *N*6-Succinyl adenosine (HR, 1.88 and 1.51), phenyllactate (HR, 2.71 and 1.79), and kynurenine (HR, 4.40 and 2.19) were associated with increased death and MACE risks (*P* < 0.05). DL-P-hydroxyphenyllactic acid with HR of 3.11, adipic acid (HR = 4.67), S-(5-Adenosy)-l-homocysteine (HR = 2.88), trimethylamine *N*-oxide (HR = 1.76), 3,3ʹ,5-Triiodo-l-thyronine (HR = 0.11), and 4-acetamidobutyric acid (HR = 2.34), were linked to the risks of death. d-Sorbitol with HR of 1.76 and phenylacetyl-l-glutamine with HR of 1.74 were linked to increased risk of MACE. Moreover, we still observed 14 metabolites, including 4-acetamidobutyric acid (estimate [SE], − 4.19 [1.18]), phenylacetyl-l-glutamine (estimate [SE], − 4.53 [1.32]), phenyllactate (estimate [SE], − 2.68 [1.09]), kynurenine (estimate [SE], − 4.24 [1.80]), adipic acid (estimate [SE], − 4.29 [2.11]), kynurenic acid (estimate [SE], − 3.55 [1.51]), 2-(dimethylamino) guanosine (estimate [SE], − 4.97 [1.48]), β-pseudouridine (estimate [SE], − 3.07 [1.35]) were associated with decreased LVEF, and Dl-2-aminooctanoic acid (estimate [SE], − 9.63 [4.53]) were associated with decreased LVMI.

Moreover, mediation analysis of LVEF was performed to further explain the associations between the metabolites and the clinical endpoints. Here, only those metabolites that were observed to have causal effects on LVEF in MR analysis were focused on. Mediation analysis proposed that the seven metabolites increase death and MACE risks could partly through impair LVEF (*P* value of Total effect and Med-eff < 0.05, Fig. 4C and Additional file 1: Table S11). The percentage of the total effect mediated by decreased LVEF on both death and MACE of β-pseudouridine was estimated at 18–20%, 2-(dimethylamino) guanosine and phenylacetyl-l-glutamine was at 18–19%, phenyllactate was at 19% (death) and 13% (MACE), kynurenine was at 18% on death and 14% on MACE, 4-acetamidobutyric acid and kynurenic acid were at 13–15%.

## Discussion

In this prospective cohort of Chinese CAD patients, we described a comprehensive metabolomic study to identify endogenous metabolites that were associated with the major clinical outcomes of CAD, i.e. death and MACE. Based on these findings, we built prognostic models and found that combining metabolic signatures and clinical factors outperformed other models that used solely clinical risk factors, or clinical factors with established biomarker TMAO. Subsequently, we performed MR analysis to assess the potential causal effects of the metabolites on clinical outcomes. We observed that 11 metabolites were causally associated with the risk of death or MACE, including kynurenine, *N*6-Succinyl adenosine, phenyllactate, DL-P-hydroxyphenyllactic acid, 3,3ʹ,5-Triiodo-l-thyronine (T3), adipic acid, S-(5-Adenosy)-l-homocysteine, TMAO, 4-acetamidobutyric acid, d-sorbitol, and phenylacetyl-l-glutamine. In addition, we proposed for the first time that 7 metabolic signatures inferred as causal on the reduced LVEF by MR analysis increased the risks of death and MACE possibly partly through impairing LV function, including kynurenine, phenylacetyl-l-glutamine, 4-acetamidobutyric acid, phenyllactate, kynurenic acid, and two modified nucleosides. To our knowledge, our study provided the most comprehensive metabolomic and causal inference for the major clinical outcomes of CAD to date, suggesting potential pathological mechanisms, and offering biomarkers and potential therapeutic targets for the secondary prevention of CAD.

Deciphering the details of the circulating metabolome could help to predict the risk of CAD and subsequent cardiovascular events [24]. To date, many studies have already identified some biomarkers for CAD risk, such as sphingolipids [25] and TMAO [26]. Moreover, prior studies identified several metabolic markers for all-cause mortality in CAD patients of European ancestry [14, 15]. However, the metabolomic biomarkers have not been well studied for clinical outcomes of CAD in Asian population especially Chinese at present [27]. In our study on Chinese CAD patients, we replicated several of them, including kynurenine, T3, S-(5-adenosy)-l-homocysteine), TMAO, and phenylacetyl-l-glutamine. Interestingly, the impacts of these metabolites on the death or MACE risk were further confirmed by our MR analysis. Their potential biological mechanisms in CAD were discussed as follows. Increased catabolism of tryptophan to kynurenine was related to cardiovascular events and mortality [28, 29], and activation of tryptophan–kynurenine pathway was correlated with LV dysfunction [30]. Consistent with this, our mediation analysis showed that 13–18% of adverse effects of kynurenine and its metabolites kynurenic acid on death and MACE risks could be explained by reduced LVEF. Kynurenine and several of its downstream can bind to transcription factor aryl hydrocarbon receptor and mediate T cell apoptosis and vascular inflammation, which in turn regulates inflammation-induced CVD [31]. Therefore, dysregulation of tryptophan metabolism represents a functional mechanism and generates biomarkers for early prognosis and therapeutic intervention. Low T3 level was associated with an increased risk of death, indicating an important role of thyroid function in CAD, which was consistent with a previous meta-analysis that low serum T3 level was related to increased risk of all-cause mortality and MACE [32]. S-(5-adenosy)-l-homocysteine) was reported as a strong predictor of mortality in cirrhosis [33], and as a more sensitive biomarker than homocysteine for cardiovascular events [34, 35].

TMAO and phenylacetyl-l-glutamine were two well-known gut microbiota-derived metabolites. TMAO is an established biomarker for cardiovascular events and mortality [36–39]. TMAO impacts multiple aspects of ‘patient vulnerability’, including atherosclerotic plaque development, platelet hyperresponsiveness [37, 40], and variation in macrophage and endothelial cell phenotype [41]. More recently, phenylacetyl-l-glutamine began to attract attention as a novel marker for CAD [42] and other cardiovascular diseases [43]. It was derived from gut microbiota, and via adrenergic receptors, it could enhance platelet responsiveness and athero-thrombosis, resulting in cardiovascular events. Here we reported for the first time that phenylacetyl-l-glutamine increased the risks of death and MACE in CAD could be explained at 18–19% by reduced LVEF, indicating a novel mechanistic link to cardiovascular events. The phenylacetyl-l-glutamine is regarded as a predictor of the event of heart failure [44], the accumulation of phenylacetylglutamine in heart failure may be due to increased amino acid degradation. Taken together, these findings support the notion that gut microbiota-derived metabolites are closely associated with cardiovascular health, and provide clues for developing novel therapeutic strategies in the cardiovascular field.

In addition to the known metabolic markers, we discovered additional markers that were not reported in previous CAD studies yet were revealed through our comprehensive study design of recruiting patients from multiple clinical centers. These novel markers included modified nucleosides, aromatic lactic acids, 4-acetamidobutyric acid, etc. The finding that increased plasma levels of modified nucleosides in CAD patients at high risk of major clinical outcomes is intriguing. Two modified nucleosides, 2-(dimethylamino) guanosine and β-pseudouridine, were found in our study as robust metabolic signatures for high risks of death and MACE, despite no causal relationship being observed in the MR analysis. Importantly, they displayed the strongest correlation with LV remodeling and were causally associated with reduced LVEF in MR. Furthermore, mediation analysis also inferred that their associations with clinical outcomes could be explained about 18–20% through impaired LVEF. It was consistent with recent studies that increased levels of 2-(dimethylamino) guanosine and β-pseudouridine levels were related to incident heart failure and LV remodeling [45, 46]. Furthermore, 2-(dimethylamino) guanosine has been linked to increased risk of coronary artery calcium [47] and all-cause mortality in diabetes [48], and elevated circulating levels of β-pseudouridine were associated with atrial fibrillation [49], heart failure [50] and CAD [51]. Here we report for the first time that β-pseudouridine was also a metabolic marker for the risks of death and MACE in CAD. Moreover, increased circulating modified nucleosides were also associated with the development of pulmonary hypertension, reflecting elevated stress and increased proliferation of pulmonary vascular cell [52]. Modified nucleosides consist of various RNA species, reflecting the upregulation of translation and hypercatabolism in general [53]. Taken together, our findings suggested that CAD patients may experience disorders of modified nucleoside, causing excessive damaging stress in cardiomyocytes, which in turn promoted cardiac remodeling and major clinical outcomes.

4-Acetamidobutyric acid appeared in our study as the metabolite most strongly associated with both risks of death and MACE, and about 14–15% of these effects could be mediated by promoting LV malfunction. Recent studies also found that 4-acetamidobutyric acid was associated with incident heart failure and LVMI [46], rheumatoid arthritis [54], and severity of liver and kidney disease in cirrhosis [33]. 4-Acetamidobutyric acid is a product of polyamine, arginine and proline metabolism. Polyamines play important roles in cell proliferation, apoptosis, and tumor growth, thus serving as a target for cancer prevention and treatment [55]. Polyamine stress response could be activated by acute ischemia, leading to polyamine accumulation and cardiac cell death [56, 57]. The primary pathophysiological mechanisms of polyamines during ischemia/reperfusion injury and cardiac failure were in regulating cardiomyocyte death. Moreover, inhibition of polyamine biosynthesis could protect cardiac cells from norepinephrine‐mediated apoptosis [58]. Therefore, we speculated that 4-acetamidobutyric acid is a key polyamine underlying the progression of heart failure and CAD, which warrants further investigation.

In addition, it is interesting to note that two elevated aromatic lactic acids, phenyllactate, and DL-P-hydroxyphenyllactic acid, were inferred being causally associated with the adverse outcomes in CAD. They have been reported to be produced by the human gut microbiome [59] and were positively associated with hepatic steatosis [60], cirrhosis [33], and hepatocellular carcinoma development [61]. Recent studies reported that 3-(4-hydroxyphenyl) lactacte significantly increased the risk of diabetes [62], and in rodents, hydroxyphenyllactate may decrease the production of reactive oxygen species in mitochondria and neutrophils [59]. While their biological linkage with CAD and heart failure needs further study. *N*6-succinyl adenosine, a purine metabolite, was reported to accumulate in the fluid of patients with adenylosuccinase deficiency, causing severe neurological impairment [63]. *N*6-succinyladenosine was reported to promote inflammasome activity and IL-1β production in subjects with high expression of the inflammasome module, which was related to all-cause mortality [64]. Moreover, *N*6-succinyl adenosine was dramatically increased following myocardial infarction [65] in rats and significantly elevated in patients with chronic thromboembolic pulmonary hypertension [66], providing clues to the association with CAD. Adipic acid is a product of lipid oxidation and could predict the development of islet autoantibodies [67]. A study reported that accumulated adipic acid as toxic metabolites from medium-chain acyl-CoA dehydrogenase deficiency-induced significant DNA damage in vitro [68].

Understanding the effects of these metabolic markers on the clinical events to be causal or merely associated could shed light on potential novel intervention targets for CAD. Therefore, unique in this study, we have explored whether there are potential causal effects of metabolic markers on the prognosis and LV remodeling of CAD using MR analysis. MR analysis is a popular causality inference tool that uses the inherent genotype as the instrumental variable which follows Mendelian laws of inheritance [65]. Genotypes as instrumental variables are not affected by confounding factors such as diet and other environmental factors. Hence, MR analysis could help us to rule out the influences from confounders and have causal timing, thus providing unbiased estimates of causality between exposure and outcome. However, although MR analysis and mediation analysis provided important causal inference for our findings, those newly identified metabolic markers, are still worth-well to further causality confirmation and mechanism study.

In addition, a recent study revealed that heterogeneous metabolic deviation profiles were evident even in a homogenous subgroup of acute coronary syndrome patients, which emphasizes that personalized risk stratification and preventative measures are essential in CAD [69]. Our study confirmed that combining metabolic signatures and clinical risk factors yields a better prognostic prediction for the clinical endpoints in CAD patients, compared to using metabolic signatures or clinical risk factors alone. Furthermore, it outperformed the model of the established marker TMAO plus clinical risk factors, even though the plasma level of TMAO was previously suggested as a predictor of death and MACE in CAD [17, 36]. It was also reported that the model including TMAO, for predicting clinical endpoints resulted in limited C-statistics and no significant improvement compared with the traditional risk factors [36]. The progression of CAD is multifactorial with complex physiopathology, which concurrently alters multiple metabolic pathways. Therefore, a joint metabolic signature has better strength to capture the complex changes, leading to improved tools for early risk stratification for the secondary prevention of CAD. Future investigations will help evaluate and improve the clinical utility of our model for different patient populations.

## Limitations

First, the study was based on Chinese patients, and the sample size of the validation cohort was relatively small, therefore we only validated the results with strong signals and could miss the weak signals that were weak. Studies expanding to other ethnicities and large-scale cohorts were also needed. Secondly, due to the metabolomic platform and method updating, the conditions for metabolite detection between the two cohorts were different, leading to 600 metabolites being detected in the multicenter validation cohort, while only 160 metabolites were identical to the discovery cohort. If more metabolites were added, then more novel metabolic signatures could be found. Another limitation is that the effect of CAD severity on the clinical outcomes was controlled by adjusting the SYNTAX scores in the discovery cohort, but the multicenter cohorts did not eliminate this bias since the data was unavailable. Finally, even though this study is prospective research based on observational data and explored the potential causal relationship between the key metabolites with LV remodeling traits and the outcomes, further mechanistic experiments are needed to verify the biological linkage and whether the biomarkers we identified precede LV remodeling incidence or concomitant result. Future studies using improved metabolomic detection methods can leverage the analysis framework we described in this study for discovering more novel metabolic signatures and better prognostic models.

## Conclusions

This study provided new insights into the relationship between plasma metabolites and clinical outcomes and LV remodeling in CAD. The prediction model based on the metabolic signatures and clinical risk factors can significantly improve the risk stratification for death and MACE, serving as a potential prognostic tool. Several metabolic markers such as kynurenines, phenylacetyl-L-glutamine, 4-acetamidobutyric acid, and modified nucleosides may causally increase the risk of death and MACE, and these impacts appear to be partly mediated by impaired cardiac function, which still merits further study. These findings suggest important risk markers and potential therapeutic targets for the secondary prevention of CAD.

## Methods

### Study cohort

**Fig. 5:**
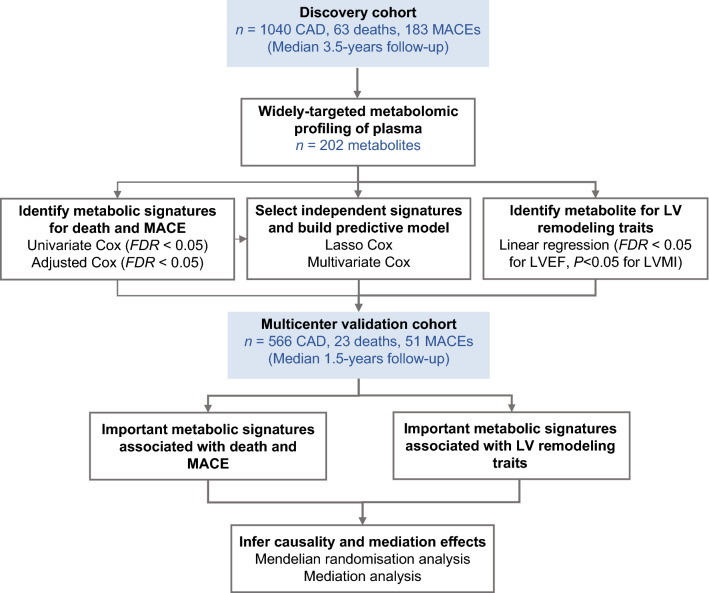
Study flow diagram

The study flow is depicted in Fig. 5. A total of 1606 Chinese patients with CAD were enrolled, including a discovery cohort and a multicenter validation cohort. Patients of the discovery cohort were sequentially enrolled from Guangdong Provincial People’s Hospital between January 2010 and December 2013 and followed up for the primary endpoint (all-cause death) and secondary endpoint (MACE) from June 2010 through April 2017 for a median of 3.5 years, with 63 deaths and 183 MACEs. Patients in the multicenter validation cohort were enrolled from Guangdong Provincial People’s Hospital (n = 354), Xiangya Hospital of Central South University (n = 178), and First Affiliated Hospital of Sun Yat-sen University (n = 34) from September 2017 to October 2018 and followed up until August 2020 for a median of 1.5 years, with 23 deaths and 51 MACEs. Most patients of this study were also included in our previous published lipidomic study [70].

Patients who were diagnosed with CAD (50% stenosis on coronary angiography) or received the percutaneous coronary intervention (PCI) were included. The exclusion criteria for patients in the discovery cohort included the following: (1) age < 18 years or > 80 years, (2) renal insufficiency (defined as serum creatinine concentration > 2 times the upper limit of normal [230 μmol/L], history of renal transplantation or dialysis), (3) hepatic insufficiency (defined as serum transaminase concentration > 2 times the upper limit of normal [80 U/L], or a diagnosis of cirrhosis), (4) being pregnant or lactating, (5) advanced cancer or haemodialysis, (6) history of thyroid problems and taking antithyroid drugs or thyroid hormone medication, (7) incomplete information about cardiovascular events during follow-up. The exclusion criteria for patients in the multicenter validation cohort was the seventh criterion above. Baseline informations, including demographics, medical history, biochemical measurements, and medication, were obtained from the hospital information database.

The primary outcome was death and the second was MACE (including death, nonfatal myocardial infarctions, coronary revascularization, and cerebral infarction). Coronary angiography was performed, and the Synergy between PCI with TAXUS and Cardiac Surgery (SYNTAX) score was calculated to assess the severity of CAD. The echocardiography was conducted to determine left ventricular ejection fraction (LVEF) and left ventricular mass index (LVMI). For detailed information see Additional file 2: Methods.

### Metabolite quantification

Widely targeted metabolomic profiling was conducted in the discovery cohort (UPLC, Shim-pack UFLC SHIMADZU CBM30A; MS, Applied Biosystems 4500 QTRAP) and the multicenter validation cohort (UPLC, Shim-pack UFLC SHIMADZU CBM30A; MS, Applied Biosystems 6500+ QTRAP) by Wuhan Metware Biotechnology, detailed information sees Additional file 2: Methods and Fig. S3. The ionisation modes and ion pairs of all metabolites see Additional file 1: Table S12. Due to the metabolomic platform and method updating, the conditions for metabolite detection between the two cohorts were different, mainly including changed MS system, ESI source operation parameters, flow rate, and injection volume. So, this led to 600 metabolites being detected in the multicenter validation cohort, while only 202 metabolites were detected in the discovery cohort, with 160 identical metabolites.

### Statistical analysis

The analytical method of this study was similar to our previous published lipidomic research [70]. Firstly, data were presented using the number (percent) for categorical variables and mean ± standard deviation (SD) for continuous variables. For metabolomic analysis, raw signals with a coefficient of variation (CV) > 50% in the quality control (QC) samples were removed, and missing values below the limit of detection were assigned to the minimum detection level for the metabolites. The CV of all metabolites see Additional file 1: Table S13. The Quality Control–Robust Loess Signal Correction algorithm was used for correction and integration to reduce the bias from a batch effect [71]. QC-RLSC is an effective way to normalize the metabolite features of the QC samples within an analytical block. Each block of the metabolomic data was scaled using Pareto scaling [72]. Detailed information to reflect our data improvement after batch correction see Additional file 2: Figs. S4 and S5.

Univariate and adjusted Cox regression analysis was performed to identify the clinical characteristics and metabolites that were associated with clinical endpoints and to estimate the hazard ratio (HR) and 95% confidence intervals (CI). Univariate and adjusted Linear regression was used to reveal the clinical characteristics and metabolites that were related to LVEF and LVMI with results presented as estimates ± standard errors (SE). In the adjusted analysis, covariates included 17 potential confounders, including age, sex, aspartate aminotransferase, estimated glomerular filtration rate, diabetes, hypertension, systolic blood pressure, glucose, cholesterol, high-density lipoprotein cholesterol, proton pump inhibitors, angiotensin-converting enzyme inhibitors, β-blockers, calcium channel blockers, current smoking, family history of cardiovascular disease (CVD) and SYNTAX. A two-tailed *P* of 0.05 was considered for statistical significance, and Benjamini–Hochberg method was used to control the false discovery rate (*FDR*) for correcting the number of metabolites in multiple hypothesis testing.

Time-to-event free survival was shown using Kaplan–Meier curves, and the *P*-values were analyzed using the log-rank test. The Spearman correlations in the metabolites associated with death and MACE risks in the adjusted analysis, together with traditional clinical factors (age, pro-brain natriuretic peptide, blood lipids, LVEF, LVMI, SYNTAX, glucose, blood pressure), correlations above the significant level of *P* < 0.01 and |rho|> 0.1 for clinical factors and |rho|> 0.2 for metabolites, were visualized using Cytoscape (version 3.7) to construct a correlation network. The metabolites with *FDR* < 0.05 in the adjusted Cox regression analysis were used for least absolute shrinkage and selection operator (lasso) Cox regression analysis (“glmnet” package) to reduce the dimensions of variables and select the powerful metabolic features to build a multivariable prognostic model. This procedure was performed within a tenfold cross-validation framework (200 repeats), and the variables with repeats zero were removed and the metabolites left were selected to establish the prognostic model using multivariate Cox regression, based on the Akaike Information Criterion (AIC) with a forward and backward stepwise process.

To evaluate the prediction efficiency of the multivariate model, hazard estimates of individuals were calculated using the following formula: h (X) = exp (β_1_X_1_ + β_2_X_2_ + …… + β_i_X_i_), where β is the regression coefficient, and X_i_ is the selected marker. Time-dependent receiver-operating characteristic (ROC) analysis (by ‘timeROC’ package in R) was used to assess the prognostic abilities of the hazard models. The model performance was assessed by calculating the area under the curve (AUC), continuous net reclassification indices (continuous NRI by ‘survIDINRI’ package in R), and integrated discrimination improvement (IDI by ‘survIDINRI’ package in R). The sensitivity and specificity of each model were calculated, and the corresponding optimal cut off values (Youden’s index) were determined according to the formula: sensitivity + specificity − 1 [73]. The multicenter validation of the predictive model was performed in another multicenter cohort based on the individually hazard estimates calculation, and the hazard stratification amongst the low (< Q1), middle (≥ Ql and ≤ Q3), and high (> Q3) hazard estimate groups were shown using Kaplan–Meier curves.

Statistical analyses were performed using GraphPad Prism 7 and R (version 4.1.0, http://www.R-project.org/).

### Mendelian randomisation analysis

In secondary analyses, we performed a one-sample MR analysis to infer the potential causal relationships between the metabolites and the outcomes of CAD as well as indicators of LV remodeling (LVEF and LVMI). A schematic diagram sees Additional file 2: Fig. S6.

Association results between genotypes and the metabolites were obtained from our previous metabolome-based genome-wide association study [74], and the results used in this study were provided in Additional file 1: Table S14. The association analysis between genotypes and LV remodeling was subjected to a linear model based on additive mode, adjusting for age, sex, aspartate aminotransferase, estimated glomerular filtration rate, antihypertensive drugs medication, hypertension, diabetes, and the first ten principal components. For each association analysis, we adopted a commonly used *P* < 1 × 10^–5^ as a threshold [75, 76] to select the single nucleotide polymorphisms (SNPs) in the discovery cohort [74] for the following analyses. Linkage disequilibrium (LD) analysis was conducted to retain the SNPs with the lowest *P*-value and the independent SNPs with them (LD r^2^ < 0.001 in a 10,000 kb window or two SNPs beyond 10,000 kb) as the independent instruments. A two-stage least squares (2SLS) method was used in this MR analysis process and we calculated the 2SLS regression in R (version 3.6.3).

Briefly, in the first stage, we regressed by exposure (X) to the instrumental variable (IV) to derive fitted values for exposure to IV (G-X). In the second stage, we regressed the outcome (Y) based on the fitted values from the first stage regression (X–Y). The causal estimated effect size is the regression coefficient for this second stage that reflects the change in outcome due to a unit change in exposure. In the case of multiple IVs, the 2SLS estimator can be viewed as a weighted average of the ratio estimates calculated from a single instrumental variable, with weights determined by the relative strength of the instrumental variable in the first stage regression. The outcome was regressed over the predicted values of exposure by using Cox regression to estimate HR and 95% CI for risks of death and MACE, using Linear regression to calculate estimates and SE for LVEF and LVMI, *P*-value < 0.05 was used to define causality.

### Mediation analysis

Mediation analysis decomposes the total exposure-outcome effect into a direct effect and an indirect effect through a mediator variable [77, 78]. The mediation analysis models [79] were constructed to examine whether the association of metabolic signatures and risks of death and MACE could be mediated through their role in promoting LV remodelling and estimate mediation effects size. A schematic diagram sees Additional file 2: Fig. S6. Metabolites were continuous predictor variables (X); LVEF were continuous mediators (M); death and MACE were dichotomous outcome variables (Y). In this study, we performed the mediation analyses in the following four steps: (1) regressing outcomes (death and MACE, respectively) on predictors (model 1 Y = cX), where c is total effect; (2) regressing mediators (LVEF) on predictors (model 2 M = β_1_X), where β_1_ is indirect effect 1; (3) regressing outcomes on mediators (model 3 Y = β_2_M), where β_2_ is indirect effect 2; (4) regressing outcomes (death and MACE, respectively) on predictors controlling for mediators (LVEF). The regression equation is Model Y = β_2_M + cʹX, where β_2_ is indirect effect 2, and c′ is direct effect; and calculating mediation effect as (β_1_ × β_2_)/c. Mediation analyses were conducted using Lavaan package of R 4.0.5.

## Supplementary Information

**Additional file 1: Table S1.** Baseline characteristics on death and MACE risks. **Table S2.** Baseline characteristics on LVEF and LVMI. **Table S3.** Relationship between metabolites and death risk. **Table S4.** Relationship between metabolites and MACE risk. **Table S5.** Multivariate Cox proportional hazards model for clinical outcomes. **Table S6.** Spearman correlation analysis between clinical factors and those metabolites associated with death risk. **Table S7.** Spearman correlation analysis between clinical factors and those metabolites associated with death risk. **Table S8.** Relationship between metabolites and LVEF. **Table S9.** Relationship between metabolites and LVMI. **Table S10.** Estimation of the potential causal relationships between the metabolites and outcomes and LV remodeling traits by MR analyses. **Table S11.** Mediation effect of metabolites-LVEF-outcomes association. **Table S12.** The ionisation modes and ion pairs of the metabolites. **Table S13.** The coefficient of deviation of the metabolites between QC samples. **Table S14.** Associations between SNP and metabolites in the metabolome-based genome-wide association study.

**Additional file 2.** Additional Methods. **Fig. S1.** The Kaplan–Meier curves of LVEF (A-B) and LVMI (C-D) for risks of death and MACE in the discovery cohort. **Fig. S2.** Correlation network of metabolic signatures for MACE risk and clinical factors. **Fig. S3.** Representative total ion flow diagrams between different QC samples under positive ion mode (A) and negative ion mode (B). **Fig. S4.** The visualization image analysis of the quality improvement procedures for typical features (Kynurenine) in metabolomics data. **Fig. S5.** Comparison of the cumulative frequency of RSD% of all features in QC samples before and after batch correction by QC-RLSC. **Fig. S6.** Diagram of Mendelian randomization analysis and mediation analysis.

## Data Availability

The datasets used and/or analysed during the current study are available from the corresponding author on reasonable request.
