# Correction: Plasma metabolomics provides new insights into the relationship between metabolites and outcomes and left ventricular remodeling of coronary artery disease

**DOI:** 10.1186/s13578-022-00926-z

**Published:** 2022-12-16

**Authors:** Qian Zhu, Min Qin, Zixian Wang, Yonglin Wu, Xiaoping Chen, Chen Liu, Qilin Ma, Yibin Liu, Weihua Lai, Hui Chen, Jingjing Cai, Yemao Liu, Fang Lei, Bin Zhang, Shuyao Zhang, Guodong He, Hanping Li, Mingliang Zhang, Hui Zheng, Jiyan Chen, Min Huang, Shilong Zhong

**Affiliations:** 1 Department of Pharmacy, Guangdong Provincial People’s Hospital, Guangdong Academy of Medical Sciences, Guangzhou, 510080 Guangdong China; 2 Guangdong Provincial Key Laboratory of Coronary Heart Disease Prevention, Guangdong Cardiovascular Institute, Guangdong Provincial People’s Hospital, Guangdong Academy of Medical Sciences, Guangzhou, 510080 Guangdong China; 3 School of Medicine, South China University of Technology, Guangzhou, 510080 Guangdong China; 4 Department of Clinical Pharmacology, Xiangya Hospital, Central South University, Changsha, 410008 Hunan China; 5 Department of Cardiology, The First Affiliated Hospital of Sun Yat-Sen University, Guangzhou, 510080 Guangdong China; 6 Department of Cardiology, Xiangya Hospital, Central South University, Changsha, 410008 Hunan China; 7 Institute of Model Animal, Wuhan University, Wuhan, 430072 Hubei China; 8 Department of Pharmacy, Guangzhou Red Cross Hospital, Jinan University, Guangzhou, 510220 Guangdong China; 9 Wuhan Metware Biotechnology Co., Ltd., Wuhan, 430000 Hubei China; 10 Institute of Clinical Pharmacology, School of Pharmaceutical Sciences, Sun Yat-Sen University, Guangzhou, 510006 Guangdong China

## Correction: Cell & Bioscience (2022) 12:173 10.1186/s13578-022-00863-x

In this article [1], during proof correction process, the correction in Figure 4c was incorrectly updated. The correct version of Figure 4c should have appeared as shown in this correction.
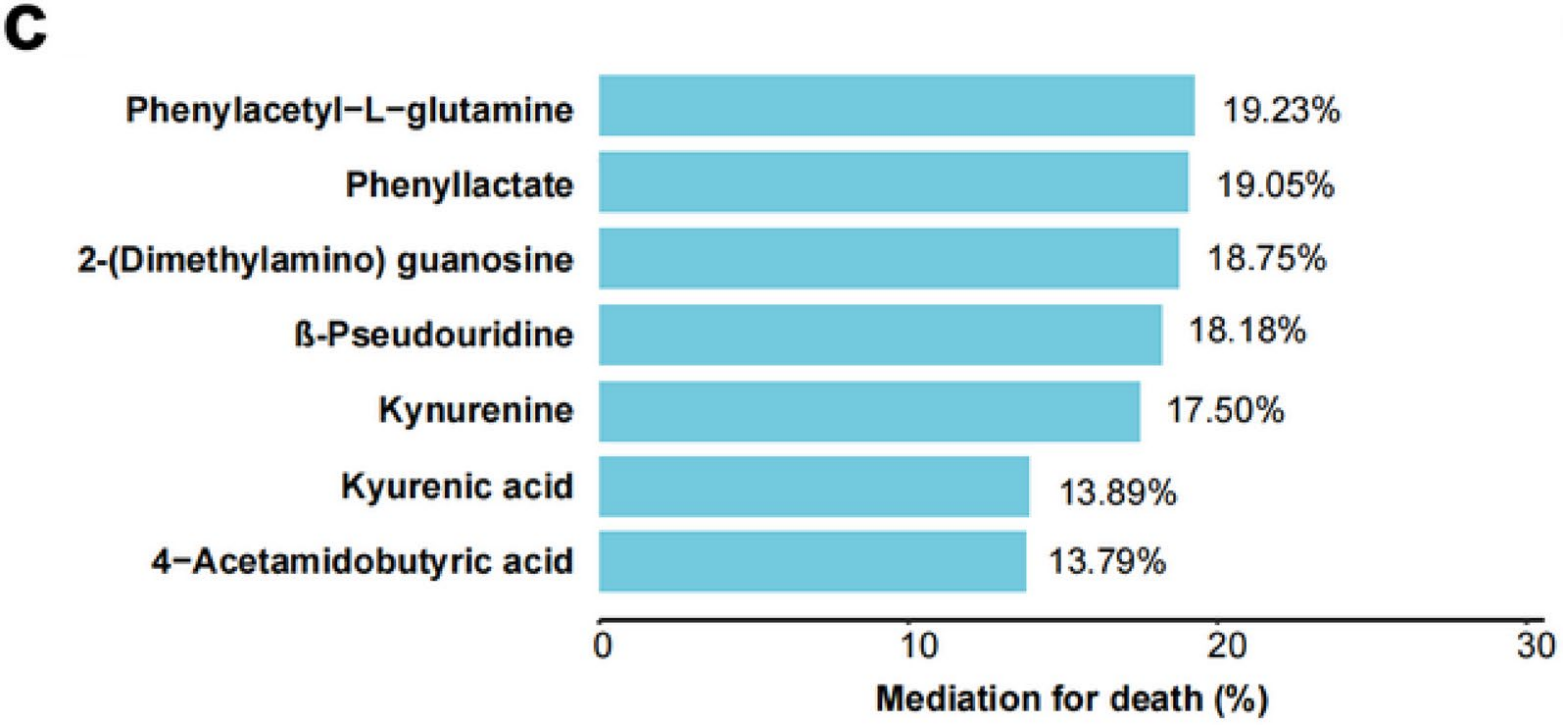


## References

[1] Zhu Q, Qin M, Wang Z, Yonglin Wu, Chen X, Liu C, Ma Q, Liu Y, Lai W, Chen H, Cai J, Liu Y, Lei F, Zhang B, Zhang S, He G, Li H, Zhang M, Zheng H, Chen J, Huang M, Zhong S (2022). Plasma metabolomics provides new insights into the relationship between metabolites and outcomes and left ventricular remodeling of coronary artery disease. Cell Biosci.

