# Supplementary material for: Plasma metabolomics provides new insights into the relationship between metabolites and outcomes and left ventricular remodeling of coronary artery disease
Source: Cell Biosci. 2022 Oct 14;12:173. doi: 10.1186/s13578-022-00863-x (PMC9569076; doi:10.1186/s13578-022-00863-x)
Supplement: Supplementary file 2 — Additional file 2. Additional Methods. Fig. S1. The Kaplan–Meier curves of LVEF (A-B) and LVMI (C-D) for risks of death and MACE in the discovery cohort. Fig. S2. Correlation network of metabolic signatures for MACE risk and clinical factors. Fig. S3. Representative total ion flow diagrams between different QC samples under positive ion mode (A) and negative ion mode (B). Fig. S4. The visualization image analysis of the quality improvement procedures for typical features (Kynurenine) in metabolomics data. Fig. S5. Comparison of the cumulative frequency of RSD% of all features in QC samples before and after batch correction by QC-RLSC. Fig. S6. Diagram of Mendelian randomization analysis and mediation analysis. [file 13578_2022_863_MOESM2_ESM.docx]

**Supplementary Information**

**Supplementary Methods**

**SYNTAX score systems**

Coronary angiography (CAG) was performed using the standard technique, and images of CAG were obtained with Syngo Dynamics cardiovascular imaging software (Siemens Medical Solutions, USA, Inc., Malvern, Pennsylvania). All angiograms were assessed by two expert cardiologists who were blinded to the metabolomic data. The SYNTAX score was used to determine the complexity and severity of CAD [[1](#_ENREF_1)]. The SYNTAX score characterizes the anatomy of coronary vasculature concerning the lesion number, lesion location, the occurrence of total occlusions, bridging collaterals, bi/trifurcations, aorto-ostial, tortuosity, lesion length, calcification, thrombus, and diffuse disease/small vessels. The SYNTAX score was calculated for each patient by using the SYNTAX score calculator version 2.11.

**Assessment of left ventricular (LV) function and structure by echocardiography**

Echocardiography was performed by two experienced cardiologists by using a Philips iE33 system (Philips Medical Systems, Bothell, WA, USA) to assess LV structure and function in accordance with the European and American Guidelines of Echocardiography [[2](#_ENREF_2)]. LV end-diastolic dimension, interventricular septal thickness, and posterior wall thickness were measured at the end diastole. LV mass (LVM) was calculated based on the Devereux formula. Body surface area (BSA) was calculated using the Stevenson formula. Thereafter, the left ventricular mass index (LVMI) was obtained by dividing LVM by BSA. LV ejection fraction (LVEF) was evaluated using the modified Simpson rule.

**Follow-up for outcomes**

The primary outcome was death and the second was MACE (including death, nonfatal myocardial infarctions, coronary revascularization, and cerebral infarction). All participants were followed up prospectively for the study endpoints based on inpatient and outpatient hospital visits and telephone contacts with the patients or their families. At each follow-up assessment (every 6 months), the participants were questioned about new adverse cardiovascular events.

**Sample collection and preparation**

Each eligible patient was fasted for at least 8 h and sampled in the morning during hospitalization, to minimize the influence of nutrition on metabolite levels. Whole blood samples were collected in EDTA-coated tubes. Blood samples were separated into the plasma and haemocyte within 2 h by centrifugation at 1000 g for 10 min at 4 °C and then stored at –80 °C for further metabolite analysis.

**Widely-targeted metabolomic profiling**

Widely-targeted metabolomic profiling was conducted in the plasma sample of the discovery and multicenter validation cohorts in March 2017 and May 2019, respectively. The widely-targeted metabolomics was performed by Wuhan Metware Biotechnology [[3](#_ENREF_3), [4](#_ENREF_4)], integrating the advantages of the “accuracy” of the targeted metabolomics and “universality” of the non-targeted metabolome, which can simultaneously quantify hundreds of known metabolites and nearly 1000 known and unknown metabolites using the innate QTRAP mass spectrometry in multiple reaction monitoring (MRM) mode, enabling high-throughput, high sensitivity, wide-coverage, and quantitative accuracy.

In the discovery cohort, for sample extraction, plasma was firstly thawed at 4 °C and then vortexed for 10 s. Then, 50 µL of plasma was transferred to 150 µL of pre-cooled methanol to precipitate proteins, vortexed for 3 min at room temperature, and then centrifuged at 12,000 rpm for 10 min at 4 °C. Thereafter, the supernatant was centrifuged again at 12,000 rpm for 3 min at 4 °C. Finally, an aliquot of the resulting supernatant was used for metabolomic analysis. Metabolomic profiling was conducted on an LC–ESI-MS/MS system (UPLC, Shim-pack UFLC SHIMADZU CBM30A; MS, Applied Biosystems 4500 QTRAP). In total, 260 metabolites were detected, including 202 annotated metabolites, such as nucleosides, amino acids, and derivatives, organic acids and derivatives, hormones, carbohydrates, and lipids.

In the multicenter validation cohort, plasma was thawed on ice, and 150 µL of ice-cold methanol was added to 50 µL of plasma. The mixture was vortexed for 3 min and then centrifuged at 12,000 rpm for 10 min at 4 °C. The supernatant was collected and then centrifuged at 12,000 rpm for 5 min at 4 °C. Finally, the resulting supernatant was used for UPLC-MS/MS analysis. The sample extracts were analyzed using an LC–ESI-MS/MS system (UPLC, Shim-pack UFLC SHIMADZU CBM30A; MS, Applied Biosystems 6500+ QTRAP). A total of 600 metabolites were annotated following constant metabolomic platform and technology updating, including 160 metabolites identical to the discovery cohort.

The separation and ESI-Q TRAP-MS/MS detection of the sample were as follows: separation was performed in a Waters ACQUITY UPLC HSS T3 C18 column (pore size 1.8 µM, length 2.1 × 100 mm) by using a gradient solvent system of water (0.04% acetic acid) to acetonitrile (0.04% acetic acid); the gradient program was 95:5 V/V at 0 min, 5:95 V/V at 11.0 min, 5:95 V/V at 12.0 min, 95:5 V/V at 12.1 min and 95:5 V/V at 14.0 min; column temperature was held at 40 °C; flow rate was set at 0.35 mL/min and 0.4 mL/min, and the injection volumes were set at 5 μL and 2 μL in the discovery cohort and the multicenter validation cohort, respectively.

The effluent was infused to electrospray ionization (ESI)-triple quadrupole-linear ion trap (QTRAP)–MS equipped with an ESI Turbo Ion-Spray interface, LIT, and triple quadrupole (QQQ) scans operated in positive and negative ion modes. In the discovery cohort, the ESI source operation parameters were as follows: source temperature was held at 550 °C; ion spray voltage (IS) was 5500 V; ion source gas I (GSI), gas II (GSII), and curtain gas (CUR) were set at 55, 60, and 25.0 psi, respectively; the collision-activated dissociation (CAD) was high. In the multicenter validation cohort, the ESI source operation parameters were as follows: source temperature was 500 ℃; IS was 5500 V (positive) and -4500 V (negative); and GSI, GSII, and CUR were set at 55, 60, and 25.0 psi, respectively; the CAD was high. Instrument tuning and mass calibration were performed with 10 and 100 μmol/L of polypropylene glycol solutions in QQQ and LIT modes, respectively. QQQ scans were acquired as MRM experiments with collision gas (nitrogen) set to 5 psi. Individual MRM transitions of declustering potential (DP) and collision energy (CE) were performed with further DP and CE optimization, and each ion pair was scanned and detected based on the optimized DP and CE. At each period, a specific set of MRM transitions was monitored based on the metabolites eluted during this period.

**Qualitative and quantitative analysis**

Qualitative analysis of the precursor ion and fragments spectra detected was carried out based on [self-built](javascript:;) MWDB (Metware database) with retention time and ion pairs, as well as the public database of metabolites information. We used MS/MS spectra to search against public databases to improve confidence in metabolite identification. Some of these substances are qualitatively analyzed with removing isotopic signals, repetitive signals containing K^+^ ions, Na^+^ ions, and NH4^+^ ions, and repeated signals of fragmented ions that themselves are of larger molecular weight. Metabolite structure resolution is referenced in existing mass spectrometry public databases such as MassBank (<http://www.massbank.jp/>) [[5](#_ENREF_5)], HMDB (<http://www.hmdb.ca/>) [[6](#_ENREF_6)] and METLIN (<http://metlin.scripps.edu/index.php>) [[7](#_ENREF_7)]. Metabolite identification was conducted by alignment of the reference standards in our [self-built](javascript:;) database and public databases, and more information is listed in **Additional file1: Table S1**.

Quantitation of metabolites was accomplished using multiple reaction monitoring (MRM) of triple quadrupole mass spectrometry. Under the MRM mode, the quadrupole rod first screened precursor ions (parent ions) of the target substance to exclude ions corresponding to other molecular weight substances and to preliminarily eliminate the interference. The precursor ions were induced to ionize in the collision cell to form many fragment ions, fragment ions. The fragment ions were then filtered through the triple four-stage bar to select the necessary characteristic fragment ion needed, which eliminated the interference of non-target ions, thereby making the quantification of better accuracy and repeatability. The mass spectrum data were processed by Analyst 1.6.3 software (AB Sciex). After obtaining the metabolite spectrum analysis data of different samples, integration of peak areas was performed for the mass spectrum peaks, and the integral correction was performed for the mass spectrum peaks of the same metabolite in different samples.

**Quality control**

Quality control (QC) samples were prepared using pooling aliquots of each of the individual plasma samples, which were extracted as described above. During instrumental running, one QC sample was inserted into every 10 samples to monitor the analysis of reproducibility. The extracts of this pooled plasma sample were injected to access process variability. As an additional QC, water aliquots were extracted as part of the sample set to serve as process blanks for artifact identification. By overlapping display analysis of the total ion chromatogram (TIC) of different QC samples for mass spectrometric detection and analysis, the repeatability of the extraction and detection of metabolites can be judged, that is, technical repeatability. The high stability of the instrument ensures the repeatability and reliability of the data (**Fig. S1**).

The Quality Control–Robust Loess Signal Correction algorithm was used for correction and integration to reduce the bias from a batch effect [[8](#_ENREF_8)]. QC-RLSC is an effective way to normalize the metabolite features of the QC samples within an analytical block. Detailed information to reflect our data improvement after batch correction see **Additional file2:** **Fig. S2 and Fig. S3**. As shown in **Fig. S2**, quality control images for the typical features (Kynurenine) in metabolomics data showed that the features of QC and samples fell back into the approximate horizontal after correction. In the following Figure 3, we further calculated the cumulative frequency of RSD% of all features in QC samples (**Fig.3**), which showed that the cumulative frequency of RSD% of the signatures was significantly decreased after correction, and as much as 96.58% of metabolic signatures had decreased RSDs. The above evidence suggested that the QC-RLSC correction increases the precision of our metabolomics data well.

**References**

1. Sianos G, Morel MA, Kappetein AP, Morice MC, Colombo A, Dawkins K, et al. The SYNTAX Score: an angiographic tool grading the complexity of coronary artery disease. Eurointervention Journal of Europcr in Collaboration with the Working Group on Interventional Cardiology of the European Society of Cardiology. 2005;1(2):219.

2. Lang RM, Bierig M, Devereux RB, Flachskampf FA, Foster E, Pellikka PA, et al. Recommendations for chamber quantification. European Journal of Echocardiography. 2006;7(2):79.

3. Barberis E, Timo S, Amede E, Vanella VV, Puricelli C, Cappellano G, et al. Large-Scale Plasma Analysis Revealed New Mechanisms and Molecules Associated with the Host Response to SARS-CoV-2. Int J Mol Sci. 2020;21(22).

4. Chen W, Gong L, Guo Z, Wang W, Zhang H, Liu X, et al. A novel integrated method for large-scale detection, identification, and quantification of widely targeted metabolites: application in the study of rice metabolomics. Mol Plant. 2013;6(6):1769-80.

5. Horai H, Arita M, Kanaya S, Nihei Y, Ikeda T, Suwa K, et al. MassBank: a public repository for sharing mass spectral data for life sciences. J Mass Spectrom. 2010;45(7):703-14.

6. Wishart DS, Feunang YD, Marcu A, Guo AC, Liang K, Vazquez-Fresno R, et al. HMDB 4.0: the human metabolome database for 2018. Nucleic Acids Res. 2018;46(D1):D608-D17.

7. Smith CA, O'Maille G, Want EJ, Qin C, Trauger SA, Brandon TR, et al. METLIN: a metabolite mass spectral database. Ther Drug Monit. 2005;27(6):747-51.

8. Luan H, Ji F, Chen Y, Cai Z. statTarget: A streamlined tool for signal drift correction and interpretations of quantitative mass spectrometry-based omics data. Analytica chimica acta. 2018;1036:66-72.


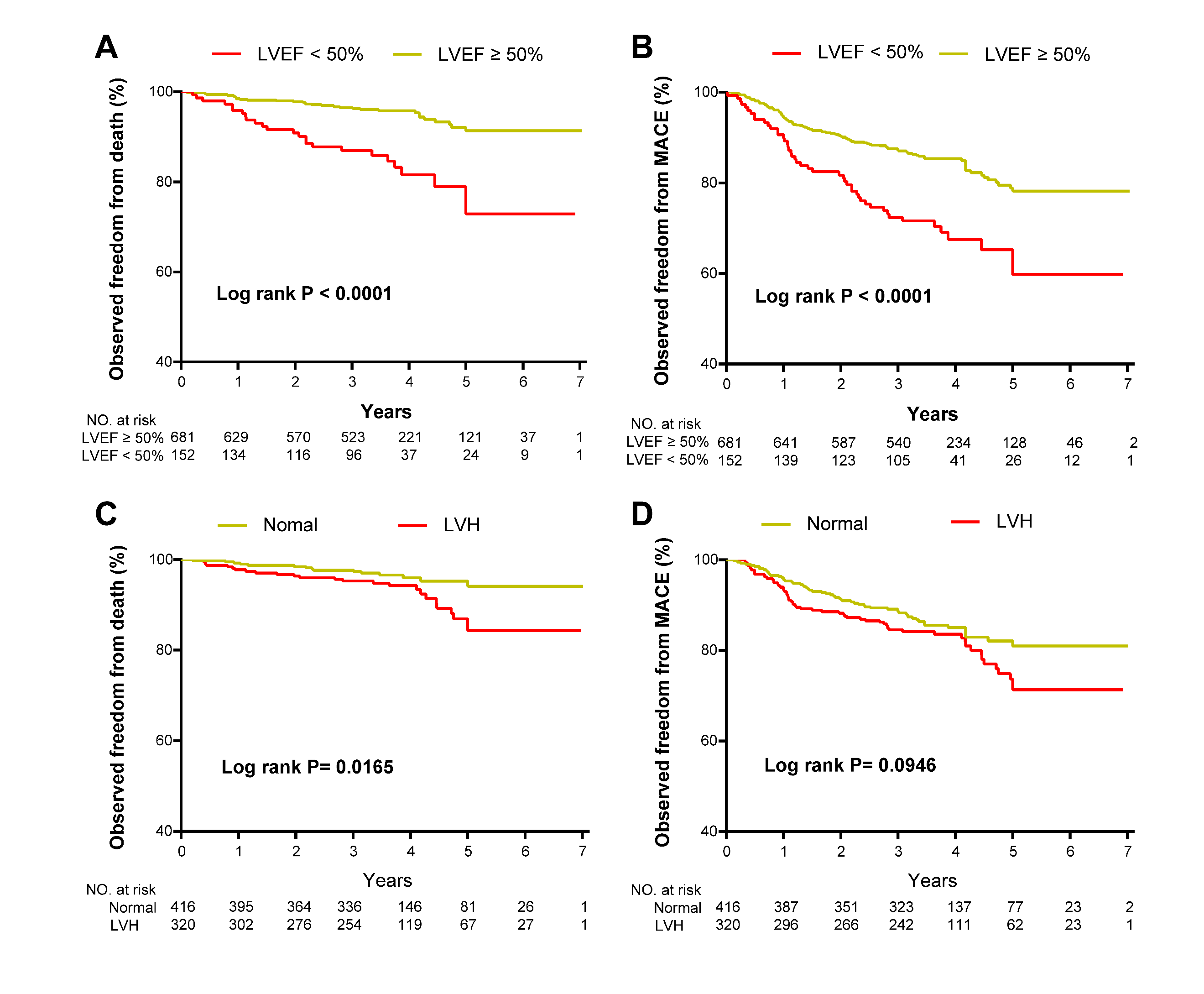


**Fig. S1 The Kaplan-Meier curves of LVEF (A-B) and LVMI (C-D) for risks of death and MACE in the discovery cohort.** *P*-values were analyzed with the log-rank test. LVH = LVMI>125g/m^2^ for male, LVMI>110 g/m^2^ for female.


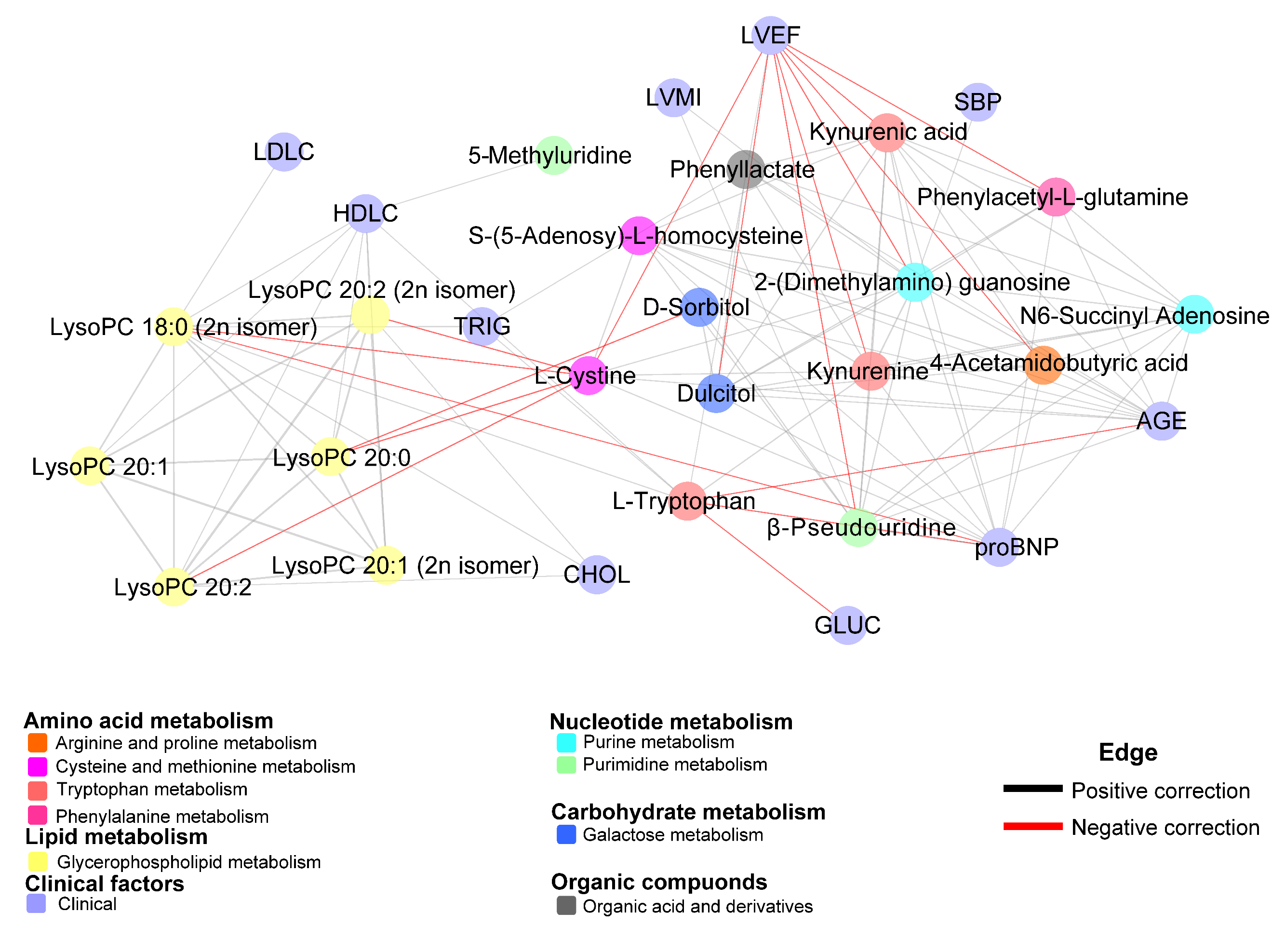


**Fig. S2 Correlation network of metabolic signatures for MACE risk and clinical factors.** Correlation network of the metabolic signatures for MACE risk in the discovery cohort and traditional clinical factors by Spearman correlations with distant nodes of |rho| >0.1 for clinical factors and |rho| >0.2 for metabolites, *P* <0.01, the rho and P-value were provided in the Table S7 of Additional file 1. Abbreviations are as Table 1.

**
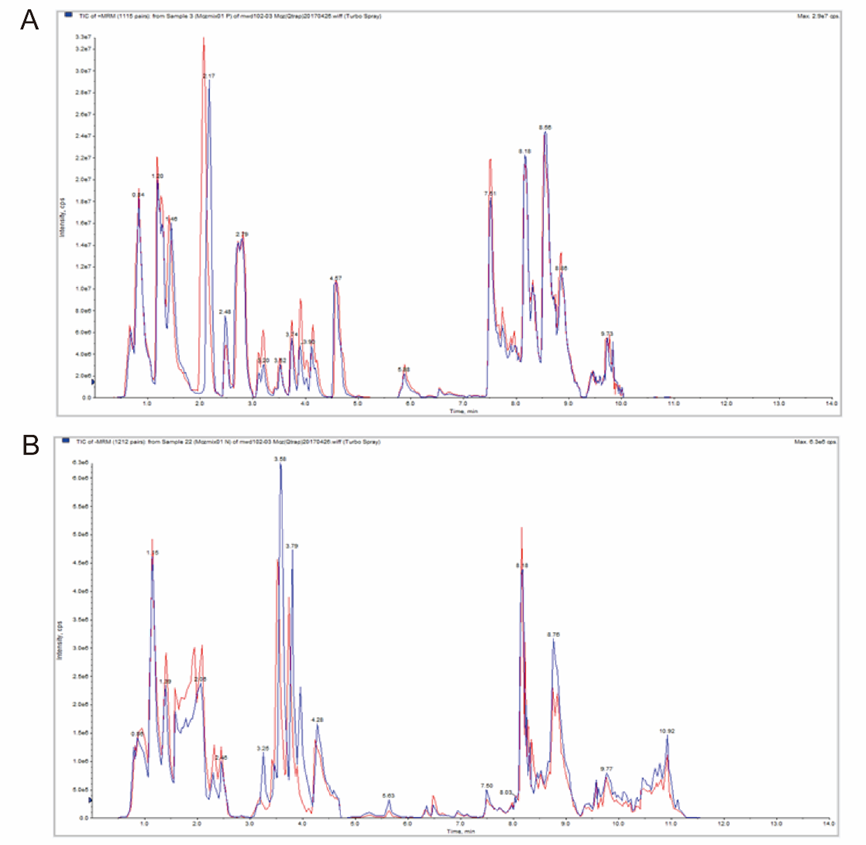
**

**Fig. S3 Representative total ion flow diagrams** **between different QC samples under positive ion mode (A) and negative ion mode (B).** Highly overlapping of the total ion chromatogram (TIC) of different QC samples for mass spectrometric detection and analysis showed the repeatability of the extraction and detection.


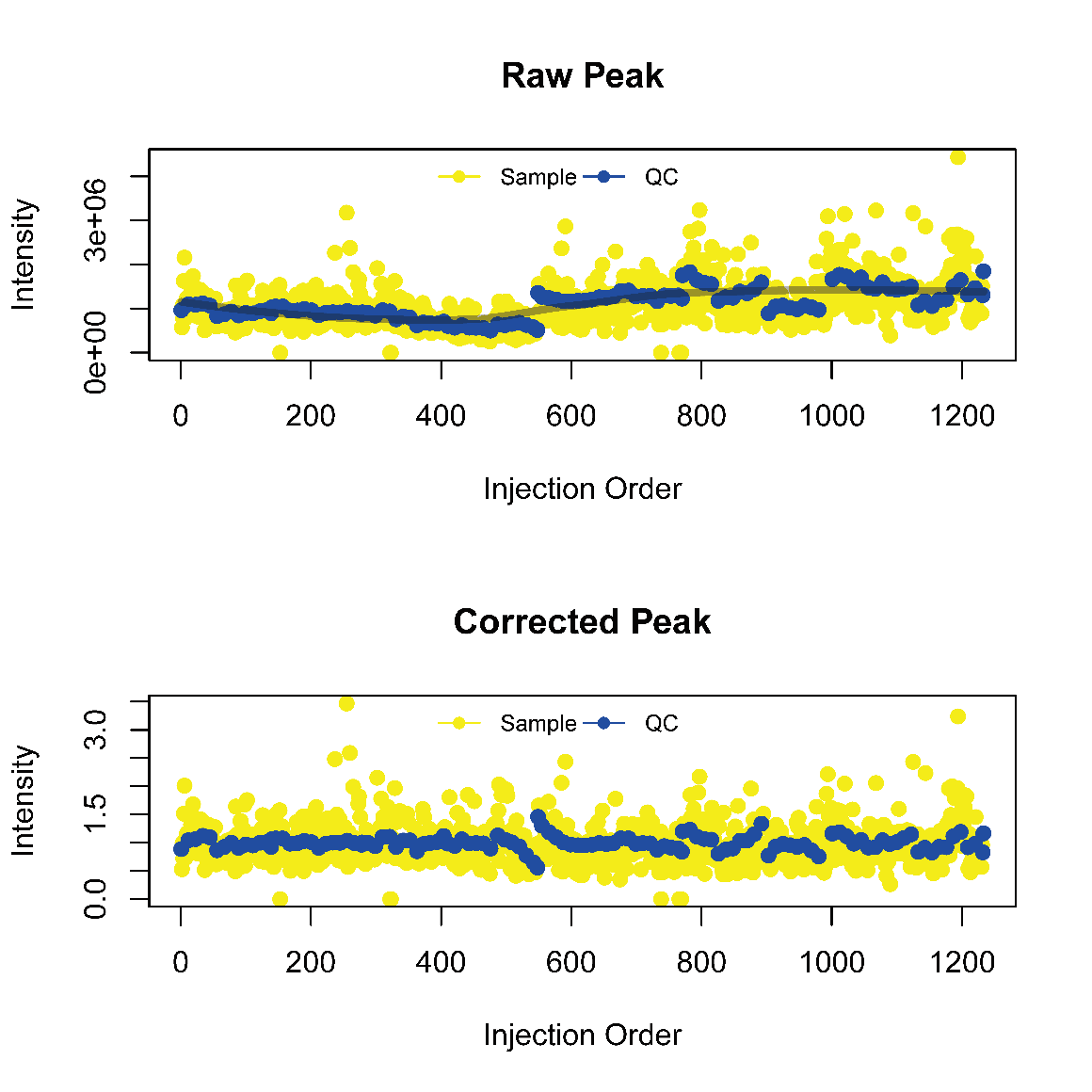


**Fig. S4** **The visualization image analysis of the quality improvement procedures for typical features (Kynurenine) in metabolomics data.**


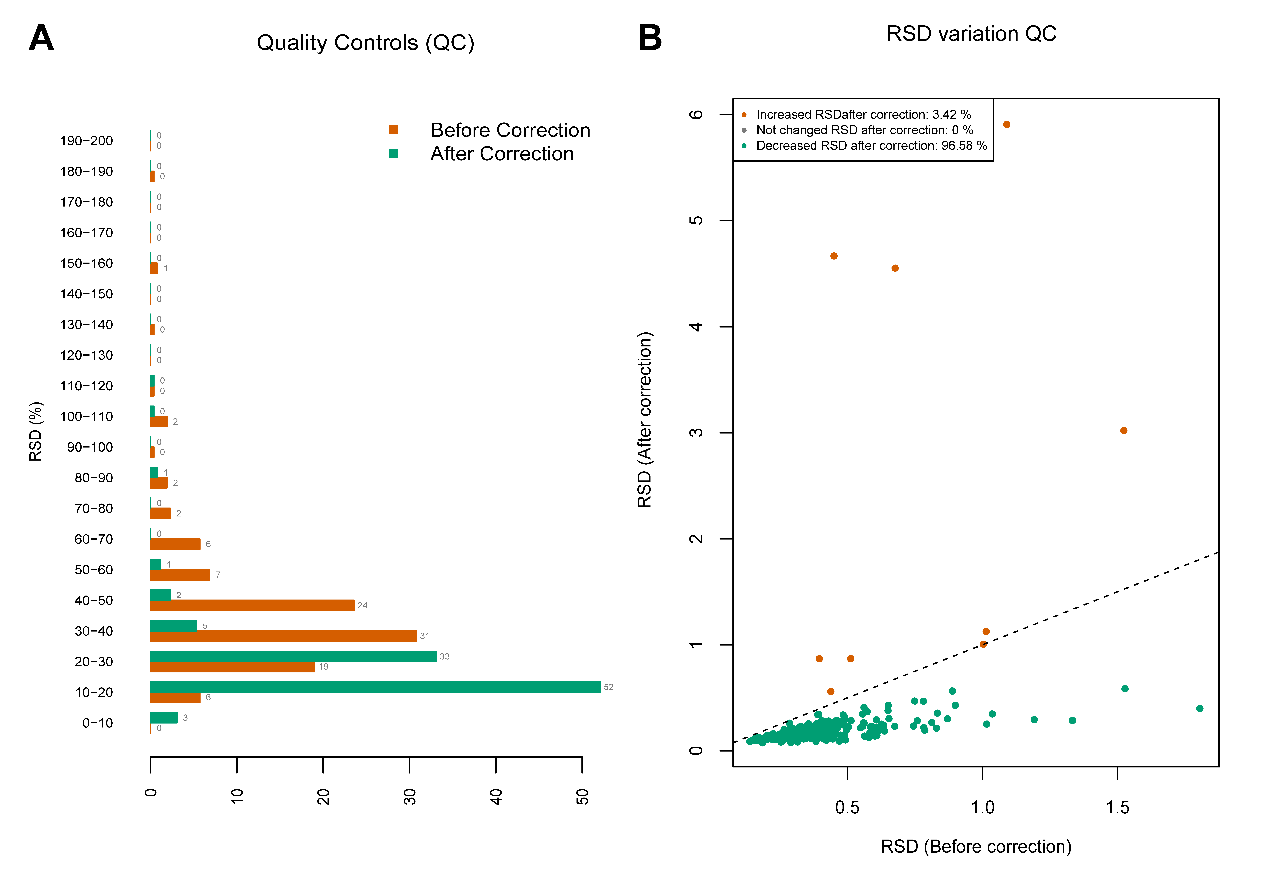


**Fig. S5 Comparison of the cumulative frequency of RSD% of all features in QC samples before and after batch correction by QC-RLSC. (A)** Histogram of RSD% distribution of features. **(B)** Scatter plot of features with decreased RSD%.


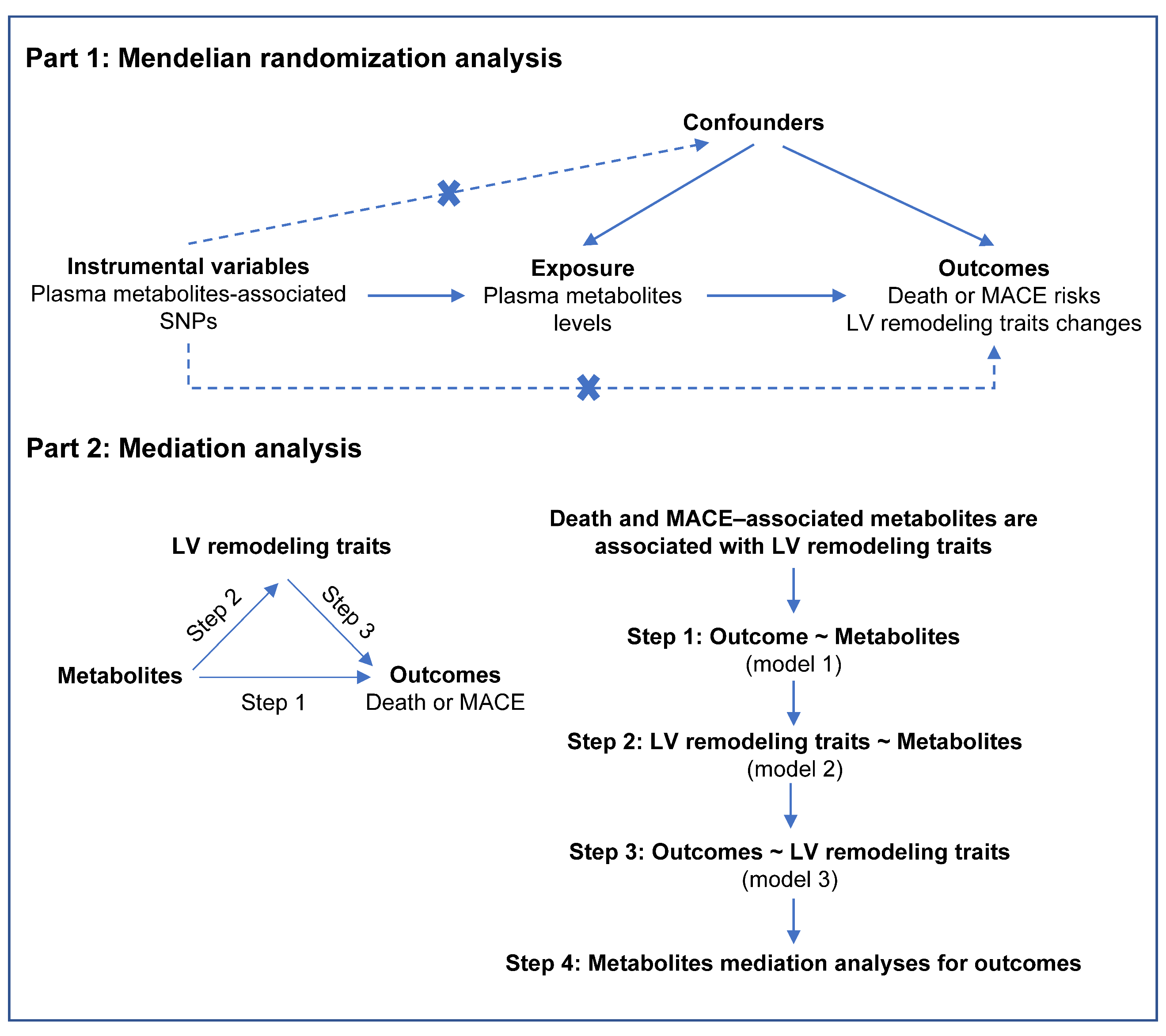


**Fig. S6 Diagram of Mendelian randomization analysis and mediation analysis. Part 1:** Schematic diagram representation of our MR analysis to test the association of plasma metabolite levels with risks of death and MACE. The diagrm shows that : 1) instrumental SNPs are associated with plsma metabolites; 2) instrumental SNPs are not associated with confounders; 3) instrumental SNPs do not have direct effects on the risk of death and MACE, i.e., their effects on death and MACE risks are mediated only through plasma metabolites. Because of the randomization of alleles at meiosis, SNPs are not associated with confoundders that may bias estimates obtained from observational studies. **Part 2:** Diagram depicting the study design of the mediation model (left) and stepwise analyzing diagram (right) for mediation analyses of LV remodeling traits in between metabolites and outcomes in CAD. Step 1, model 1 was used to analyze the association of metabolites and outcomes (death and MACE) to obtain a direct effect of predictors on outcomes. Step 2, model 2 was applied to analyze the association of metabolites and LV remodeling traits to obtain indirect effects of predictors on mediators. Step 3, model 3 was used to analyze the association of outcomes and LV remodeling traits to obtain indirect effects of mediators on outcomes. Step 4, regressing the outcome on metabolites controlling for LV remodeling traits to obtain mediation effects.
